# Effects of Orthogeriatric Care Models on Outcomes of Hip Fracture Patients: A Systematic Review and Meta-Analysis

**DOI:** 10.1007/s00223-021-00913-5

**Published:** 2021-09-30

**Authors:** Annelore Van Heghe, Gilles Mordant, Jolan Dupont, Marian Dejaeger, Michaël R. Laurent, Evelien Gielen

**Affiliations:** 1 Faculty of Medicine, KU Leuven, Leuven, Belgium; 2 Institute of Statistics, Biostatistics and Actuarial Sciences, UCLouvain, Louvain-la-Neuve, Belgium; 3 Gerontology and Geriatrics, Department of Public Health and Primary Care, KU Leuven, Leuven, Belgium; 4 Centre for Metabolic Bone Diseases, UZ Leuven, Leuven, Belgium; 5 Department of Geriatrics, University Hospitals Leuven, Herestraat 49, 3000 Leuven, Belgium; 6 Geriatrics Department, Imelda Hospital, Bonheiden, Belgium

**Keywords:** Geriatric co-management, Hip fracture, Meta-analysis, Orthogeriatrics, Osteoporosis, Systematic review

## Abstract

Orthogeriatrics is increasingly recommended in the care of hip fracture patients, although evidence for this model is conflicting or at least limited. Furthermore, there is no conclusive evidence on which model [geriatric medicine consultant service (GCS), geriatric medical ward with orthopedic surgeon consultant service (GW), integrated care model (ICM)] is superior. The review summarizes the effect of orthogeriatric care for hip fracture patients on length of stay (LOS), time to surgery (TTS), in-hospital mortality, 1-year mortality, 30-day readmission rate, functional outcome, complication rate, and cost. Two independent reviewers retrieved randomized controlled trials, controlled observational studies, and pre/post analyses. Random-effects meta-analysis was performed. Thirty-seven studies were included, totaling 37.294 patients. Orthogeriatric care significantly reduced LOS [mean difference (MD) − 1.55 days, 95% confidence interval (CI) (− 2.53; − 0.57)], but heterogeneity warrants caution in interpreting this finding. Orthogeriatrics also resulted in a 28% lower risk of in-hospital mortality [95%CI (0.56; 0.92)], a 14% lower risk of 1-year mortality [95%CI (0.76; 0.97)], and a 19% lower risk of delirium [95%CI (0.71; 0.92)]. No significant effect was observed on TTS and 30-day readmission rate. No consistent effect was found on functional outcome. Numerically lower numbers of complications were observed in orthogeriatric care, yet some complications occurred more frequently in GW and ICM. Limited data suggest orthogeriatrics is cost-effective. There is moderate quality evidence that orthogeriatrics reduces LOS, in-hospital mortality, 1-year mortality, and delirium of hip fracture patients and may reduce complications and cost, while the effect on functional outcome is inconsistent. There is currently insufficient evidence to recommend one or the other type of orthogeriatric care model.

**Supplementary Information:**

The online version contains supplementary material available at 10.1007/s00223-021-00913-5.

## Introduction

Hip fractures are the most devastating type of fragility fractures in older patients, as they contribute most to the morbidity, mortality, and economic cost associated with fragility fractures [1]. By the year 2050, the worldwide incidence of hip fractures is expected to increase by 310% for men and by 240% for women because of the aging of the population and age-related increase in fracture risk. The latter is the result of age-associated increase in the prevalence of osteoporosis and the risk of falling, with about 25% of women and 15% of men aged ≥ 80 years reporting at least one fall in the past 6 months [2, 3]. To illustrate, at the age of 90 years, the cumulative incidence of hip fractures is 32% for women and 17% for men [4]. In hip fracture patients, mortality ranges from 8 to 36% within the first year after the fracture and continues to be increased for more than 10 years thereafter [5, 6]. This long-term excess mortality is explained by the fact that older hip fracture patients are frail persons, who are at increased risk of comorbidity and functional deficits [7]. Among survivors, loss of function and mobility is profound. One year after a fracture, 40% of patients are unable to walk independently and 60% experience difficulties in at least one activity of daily living (ADL) [8].

Hip fracture patients are also at high risk of postoperative complications. Comorbidities, polypharmacy, and geriatric syndromes such as sarcopenia, dementia, delirium, and malnutrition challenge orthopedic surgeons taking care of hip fracture patients [9]. A multidisciplinary treatment approach may improve these outcomes. Orthogeriatric care is a model of systematic collaboration between orthopedic surgeons, geriatricians, and the multidisciplinary geriatric team, which focuses on older (mainly hip) fracture patients. Since 1950, different orthogeriatric care models have been developed, as opposed to the standard care consisting of (hip) fracture patients on an orthopedic ward with ‘as needed’ consult of the geriatrician at the request of the surgeon: (1) the orthopedic surgeon-led care in which the patient is hospitalized on an orthopedic ward with systematic consult of the geriatrician; (2) the geriatrician-led care with the patient on a geriatric medical ward and systematic orthopedic surgeon consult service; and (3) the integrated care model, where orthopedic surgeons and geriatricians cooperate in an integrated team on a ward with shared responsibility [10].

Some recent meta-analyses have shown that orthogeriatric care significantly reduces long-term mortality [11, 12]. However, inconsistencies persist regarding length of hospital stay [10, 11, 13] and short-term mortality [11, 12]. Furthermore, the effect on time to surgery (TTS) and functional status is infrequently evaluated [11], although some evidence exists regarding improved functional outcomes and execution of ADLs [13]. It is also unclear which orthogeriatric care model is associated with superior outcomes. In some systematic reviews (with or without meta-analysis), the integrated care model showed the lowest in-hospital mortality [10], TTS [10], and length of stay (LOS) [10, 11], while another meta-analysis showed a significant decrease in in-hospital mortality, TTS, and long-term mortality for the model with systematic consult of the geriatrician [11]. Still another meta-analysis found reduced long-term mortality in the geriatrician-led care model with systematic orthopedic surgeon consult service [12]. These data need to be interpreted with caution, because of the low number of studies in the meta-analyses, with sometimes large heterogeneity [11].

Taken together, previous studies have investigated the effect of orthogeriatric care models on outcomes of hip fracture patients. Although orthogeriatric care is increasingly recommended over usual care to improve outcomes of hip fracture patients, the evidence for some of the outcomes is conflicting, or at least limited. Furthermore, there is no conclusive evidence which orthogeriatric care model is superior. Therefore, the primary objective of this systematic review is to summarize the effect of orthogeriatric care on outcomes of hip fracture patients (LOS, TTS, in-hospital mortality, 1-year mortality, 30-day readmission rate, functional outcomes, complication rate, and total cost). The secondary objective is to investigate whether these outcomes are differentially affected by the one or the other type of orthogeriatric care model.

## Methods

### Search Methods

For the reporting of this systematic review, the Preferred Reporting Items for Systematic Reviews and Meta-analyses (PRISMA) guidelines were followed [14]. The research question was constructed using the Population, Intervention, Control, Outcome (PICO) procedure: “In older hip fracture patients, what is the effect of different orthogeriatric care models (compared to usual care or compared to each other) on a selection of outcome parameters?” A search string was developed with the keywords ‘orthogeriatric care models,’ ‘hip fracture,’ ‘femur fracture,’ and ‘osteoporosis.’ Full search strings are available in Supplementary data S1.

First, three databases (PubMed, Embase, Web of Science) were systematically searched and results were extracted from the earliest date available until August 5th, 2020. Secondly, duplicate records were removed using Mendeley. Third, the articles were hand-screened by two independent researchers, based on title and/or abstract. Subsequently, full-text articles were screened by the same researchers. A third researcher was consulted in case of disagreement. Disagreements were resolved by discussion until consensus was reached. Finally, a manual search in the reference section of the selected articles was performed to identify additional relevant articles.

### Study Selection Criteria

Randomized controlled trials (RCTs), controlled observational studies, pre/post analyses, as well as other systematic reviews and meta-analyses (as source documents to find primary studies) in English were considered eligible for inclusion in this review. Studies without a control group, letters to the editor, case reports, comments, and editorials were excluded. We included studies in persons older than 55 years, hospitalized with a recent hip fracture, while studies in persons with other fracture types, pathological hip fractures, and high traumatic injuries were excluded. Since this systematic review compares different orthogeriatric care models, only articles containing a clear description of the orthogeriatric care model and the usual care model were included. More specifically, collaboration between an orthopedic or trauma surgeon and a geriatrician was needed. Authors of several publications were contacted via e-mail to provide additional information on hip fracture outcomes when needed. In case studies described data from the same cohorts, the most recent data were included for analysis. Studies were excluded when they only reported data of which more recent results were available in another included publication of the same cohort.

### Assessment of Risk of Bias

For the quality assessment of the articles, the Cochrane Collaboration’s tool for assessing risk of bias was used, evaluating six criteria [15]. These criteria are random sequence generation (selection bias), allocation concealment (selection bias), blinding of participants and personnel (performance bias), blinding of outcome assessment (detection bias), incomplete outcome data (attrition bias), selective reporting (reporting bias), and other bias.

### Data Collection

Studies were categorized according to the type of care model: (1) geriatric medicine consult service (GCS; orthopedic surgeon-led model with patient on orthopedic ward and systematic consult of the geriatrician); (2) geriatric medical ward (GW; geriatrician-led model with patient on geriatric medical ward and systematic orthopedic surgeon consult service); (3) integrated care model (ICM; patient on ward with shared care and responsibility of orthopedic surgeon and geriatrician from admission until discharge. The patient is in a specialized orthogeriatric ward or in an orthopedic ward with the geriatrician integrated into the orthopedic team); and (4) standard of care (SOC). The extracted data included type of study, publication year, number of participants, demographic characteristics of the participants, inclusion and exclusion criteria, and country of origin. The following pre-defined outcome parameters were extracted: LOS, TTS, in-hospital mortality, 1-year mortality, 30-day readmission rate, functional outcomes, complication rate, and cost associated with the different orthogeriatric care models.

### Statistical Analysis

A meta-analysis was carried out for LOS, TTS, in-hospital mortality, 1-year mortality, 30-day readmission rate, and delirium. The analyses were performed in *R*, using primarily the metacont function from the package *meta*. Heterogeneity was assessed initially on a fixed-effect model. In light of the high heterogeneity and given the fact that the studies characteristics greatly differ in time and space, we recoursed to random-effects modeling. The variance of the random component was estimated using a Sidik–Jonkman estimator with Knapp–Hartung adjustments. Studies were considered outlying if their confidence interval (CI) did not overlap with the 95% CI of the pooled effect. The CI of the studies were constructed using Dunn’s procedure, i.e., each interval having a confidence level 1—5%/*k*, where *k* is the number of intervals constructed. The influence of each of the separate trials was tested using a leave-one-out procedure, which relies on Viechtbauer and Cheung’s cut-off for extreme values in the graph. The aim was to identify studies with important influence on the overall result. To control for publication bias, the results of the included studies are visualized in a funnel plot. Studies were excluded from the analysis if the reported data lack the necessary information that could not be obtained by addressing the authors directly. Decisions to exclude other studies were made based on the aforementioned procedure based on the CI, influence analysis, and funnel plots. Extracted data for functional outcomes, complication rate, and cost associated with the orthogeriatric care models are described with tables in a narrative analysis, since the results were very heterogeneous.

## Results

### Results of the Literature SEARCH

**Fig. 1:**
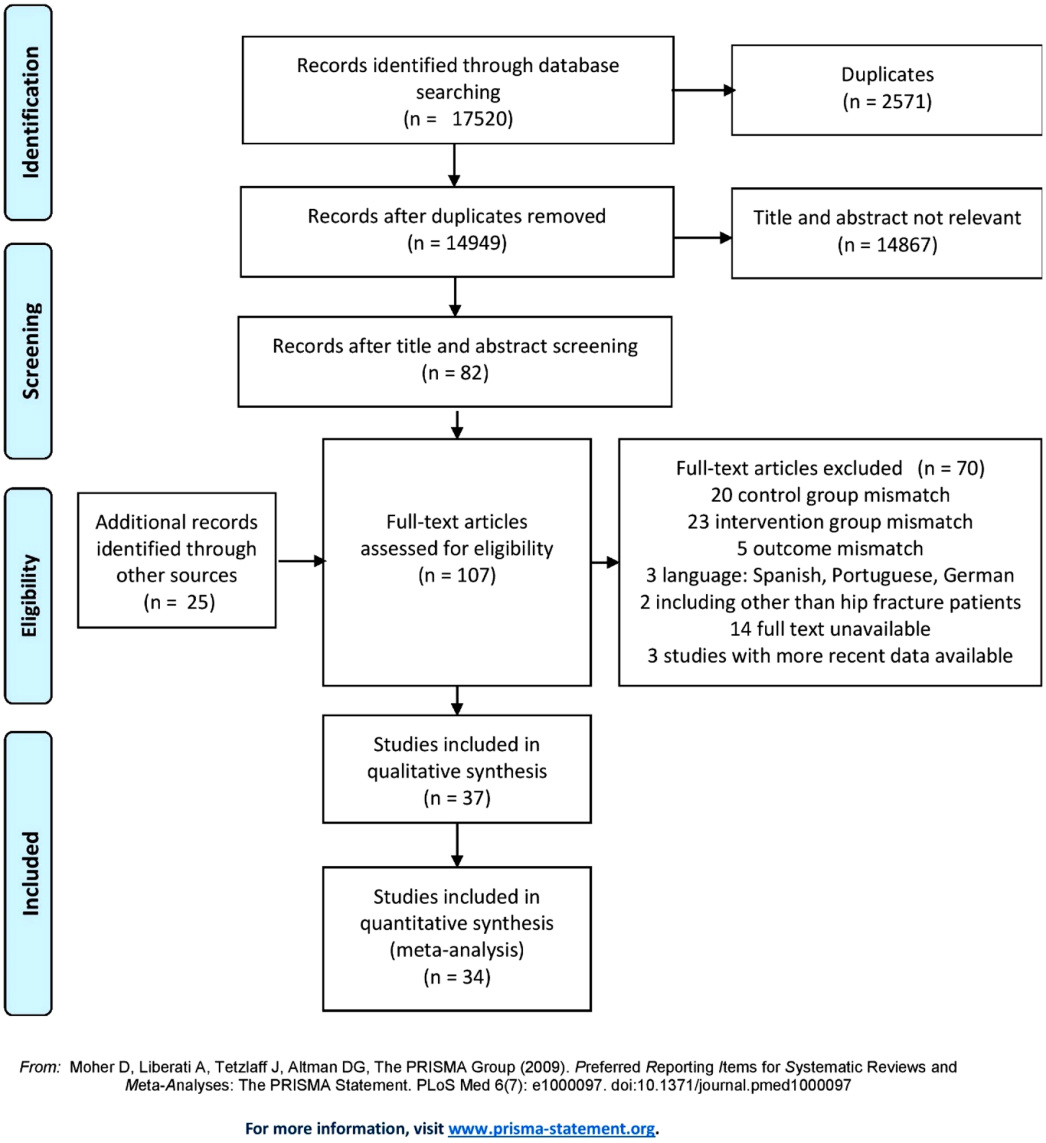
PRISMA 2009 flowchart detailing the study selection process

The PRISMA flowchart with the study selection process is shown in Fig. 1. The electronic search led to 17,520 unique records. All records were hand-screened by two independent researchers based on title and/or abstract. 14,867 irrelevant records and 2571 duplicates were identified and removed. The remaining 82 articles were read full-text and from the reference lists of these articles another 25 records were added. This resulted in 107 records that were reviewed in full-text and assessed for eligibility based on article type, study population, intervention, and outcome parameters. After applying various exclusion criteria, 37 studies were included [16–52]. Some of these studies included the same patient cohorts. This was the case for Deschodt 2011 [27] and Deschodt 2012 [28], for Adunsky 2011 [42] and Ginsberg 2013 [29], and for Prestmo 2015 [21] and Heltne 2017 [19]. When articles described the same parameters for the same cohort, the most recent data were extracted.

Table 1 summarizes inclusion criteria and study characteristics for each study. All 37 studies together included 37.294 hip fracture patients, mean age ranging from 77 to 85 years and with a majority of women in all studies. Most studies compared an orthogeriatric care model against usual orthopedic care (SOC), except in the three-arm study by Baroni et al., which compared ICM and GCS with SOC and with each other [32]. In all studies there were 13.273 patients in the intervention groups and 24.021 in the control groups. Distribution of the patients over the three orthogeriatric care models (versus control groups) was as follows: 1346 (GCS) *vs.* 1512 (SOC) in ten studies, 5383 (GW) *vs.* 8756 (SOC) in eight studies, and 6544 (ICM) *vs.* 13,963 (SOC) in 20 studies. The majority of the studies (25 out of 37) originated from European countries. The remaining 12 included articles originated from very different areas: Asia (three), USA (four), Australia (two), and the Middle-East (three).

**Table 1:**
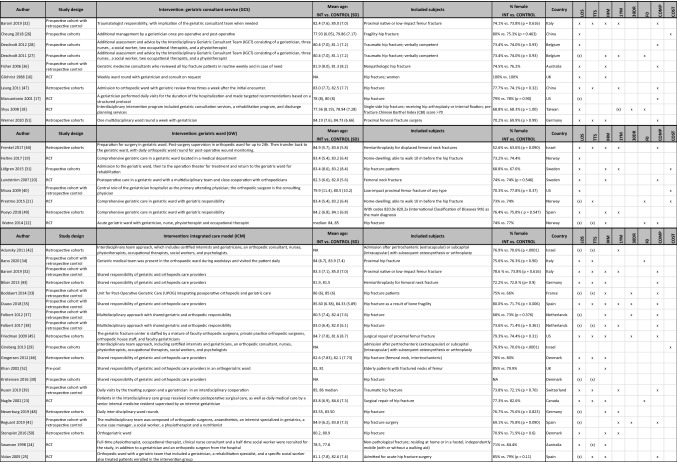
Characteristics of the included studies

*GW* geriatric ward, *GCS* geriatric consultant service, *ICM* integrated care model, *RCT* randomized controlled trial, *SD* standard deviation, *LOS* length of stay, *TTS* time to surgery, *IHM* in-hospital mortality, *1YM* one-year mortality, *30DR* 30-day readmission rate, *COMP* complication rate, *FO* functional outcome, *NA* not assessed, (x) data are available but not included in meta-analysis because more recent data from the same cohort are available and/or data do not fit the requirements for the meta-analysis

As indicated in Table 1, all studies but one [29] reported data on LOS, while data on in-hospital mortality were reported in 23 studies [16, 20, 22–25, 27, 31–33, 35–38, 43–46, 48–52]. In contrast, only about half of the studies reported data on TTS [21–25, 30, 32–35, 38, 39, 42–47, 51], complication rates [17, 20, 22, 25, 28, 32, 33, 35–39, 41, 43–45, 47, 51], and 1-year mortality [18, 27, 31, 32, 35, 38, 39, 41, 42, 44, 47–50]. 30-day readmission rate [18, 33, 35, 37, 41, 45], functional outcomes [18, 21–23, 27, 34], and cost [21, 26, 29, 31, 40] were reported in less than a quarter of the studies.

### Risk of Bias in the Included Studies

Of the 37 included studies, only ten studies were RCTs [16–25]. Six studies were prospective cohort studies [26–31], ten were prospective cohort studies with a retrospective control [32–41], ten were retrospective cohort studies [42–51], and one study was a prospective pre- and post-intervention study [52]. Results of the Cochrane collaboration’s tool for assessing risk of bias are given in Table 2. Most studies were at moderate-to-high risk of bias. Sixteen studies using a historical control group were prone to non-contemporaneous bias [32–41, 43, 46, 47, 50–52] and five studies were at high risk of bias because they compared different healthcare centers [26, 30, 45, 49] or two remote sites from the same hospital [31]. Visual inspection of funnel plots of meta-analyses (see below) revealed no asymmetry suggestive of publication bias.

**Table 2:**
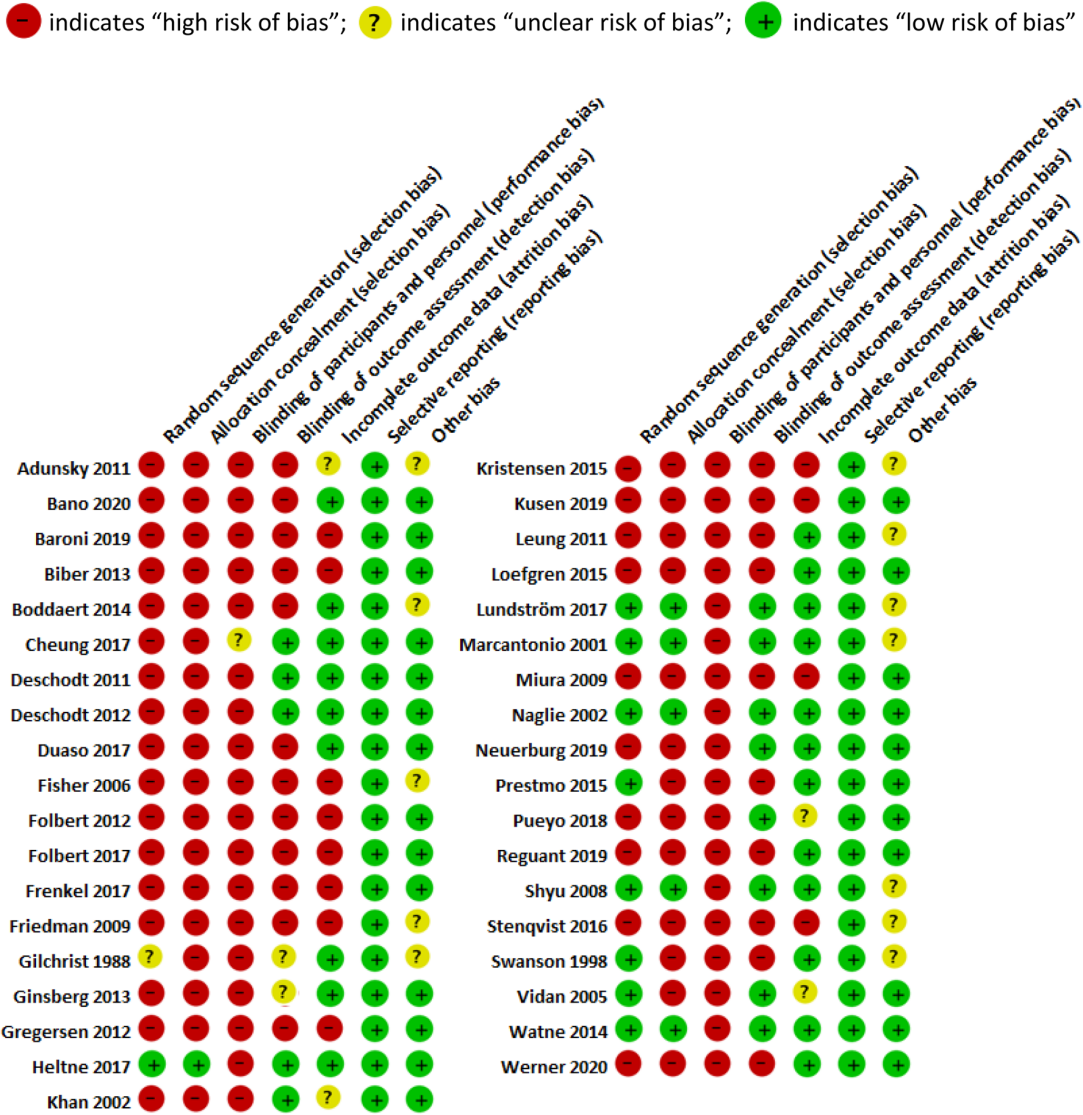
Risk of bias summary: authors’ judgements about each risk of bias for each included study

### Effects of Orthogeriatric Care on Hip Fracture Outcomes

#### Length of Stay (LOS)

**Fig. 2:**
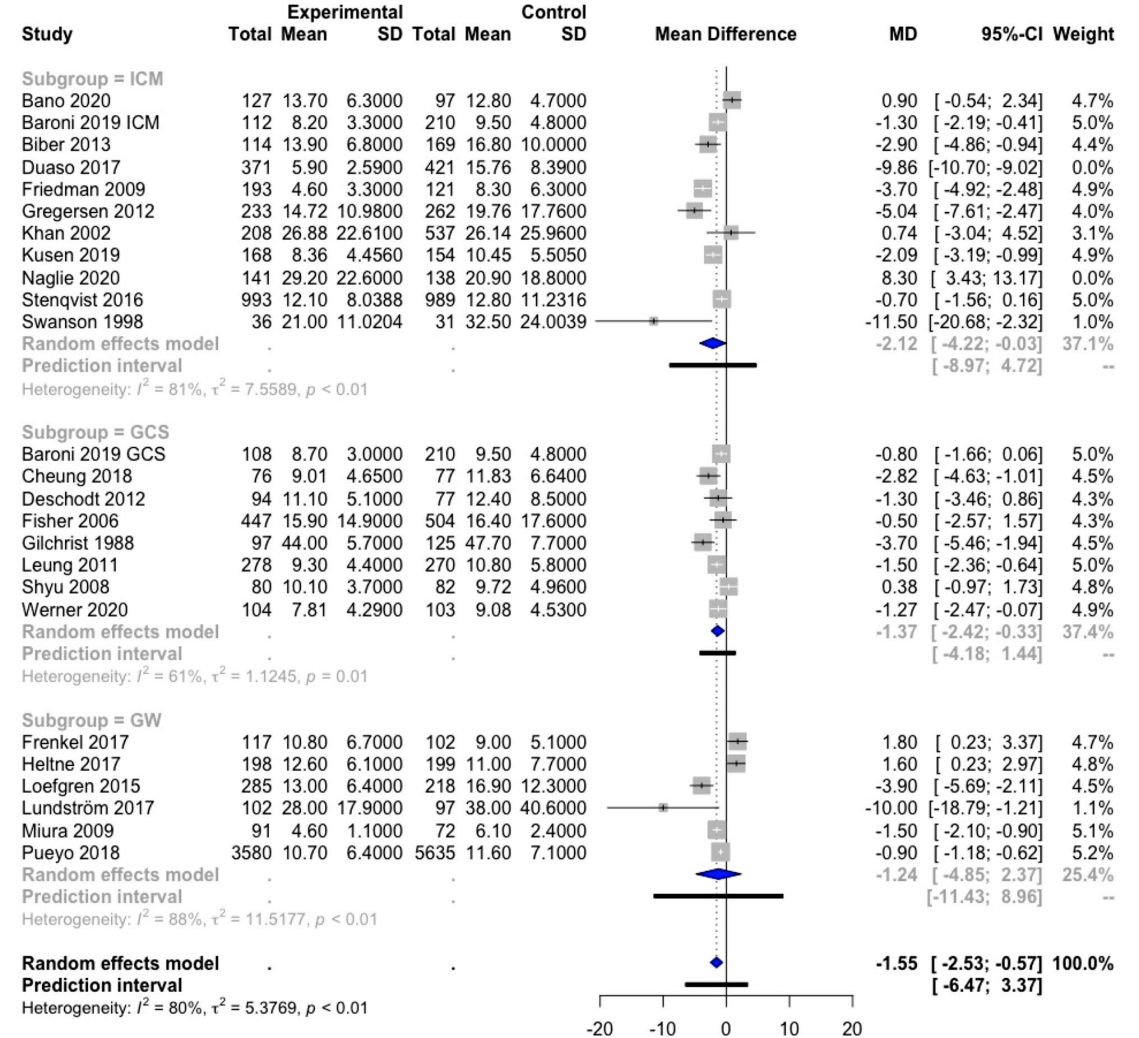
Forest plot of comparison of length of stay in hip fracture patients under orthogeriatric *vs.* usual orthopedic care. *ICM* integrated care model, *GCS* geriatric consultant service, *GW* geriatric ward, *MD* mean difference, result of Duaso et al. [35] are not included in the overall meta-analysis result nor in the ICM subgroup meta-analysis result; results of Frenkel et al. [44]and Heltne et al. [19] are not included in the overall meta-analysis result

The effect of orthogeriatric care on LOS was evaluated with a meta-analysis. Data on LOS were available in 36 studies. Ten of these could not be included in the meta-analysis as the reported data did not correspond to the data required for the meta-analysis [17, 22, 25, 30, 33, 37, 38, 41, 42, 48]. In addition, two studies were not included in the meta-analysis, as more recent data of the same cohorts were available and reported in the meta-analyses [21, 27]. As such, 24 studies (with in Baroni et al. both GCS and ICM being evaluated [32]) were included in the meta-analysis [16, 18–20, 23, 24, 26, 28, 31, 32, 34–36, 39, 40, 43–47, 49–52] (Fig. 2).

The random-effects model showed a statistically significant decrease of 1.55 days of LOS [95% confidence interval (CI) (− 2.53; − 0.57)] for all orthogeriatric care models combined compared to SOC. The heterogeneity of the results was very high (*I*^2^ = 80%), indicating that the results should be interpreted with caution. The above procedure pertaining to the CI was applied and suggested to remove the studies of Duaso et al. and Naglie et al. from the result of the meta-analysis [23, 35]. This might imply that there exist other external factors hampering the comparison with the remaining studies, which is why these two studies were not retained in the result of the meta-analysis. The studies of Frenkel et al. and Heltne et al. appeared outlying when building a global random-effects model, but not when the effect of the subgroup (GW) alone was taken into account [19, 44]. Therefore, we preferred to keep these studies in the overall and the subgroup analysis.

Considering the individual effect of the different orthogeriatric care models, the data of ICM and GW were very heterogeneous (*I*^2^ = 81% and 88%, respectively), which results in large CI [− 2.12; 95% CI (− 4.22; − 0.03) and − 1.24; 95% CI (− 4.85; 2.37), respectively]. The result of the ICM subgroup was statistically significant, whereas the result of the GW subgroup was not. However, in the GCS subgroup, there was less heterogeneity [*I*^2^ = 61%] with smaller 95% CI [− 1.37; 95% (− 2.42; − 0.33)] and the effect on LOS was significantly different from the control group.

#### Time to Surgery (TTS)

**Fig. 3:**
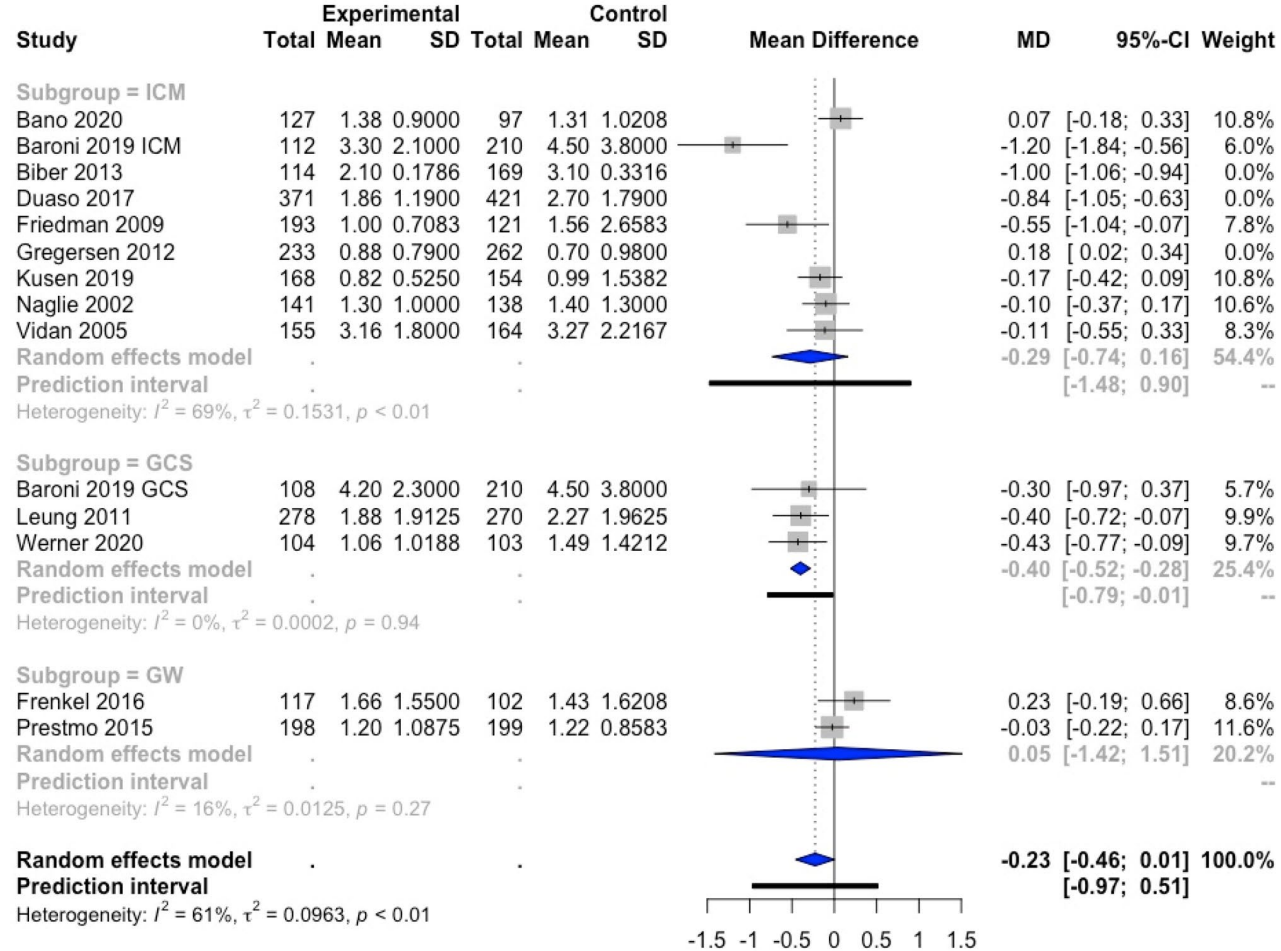
Forest plot of comparison of time to surgery in hip fracture patients under orthogeriatric *vs.* usual orthopedic care. *ICM* integrated care model, *GCS* geriatric consultant service, *GW* geriatric ward, *MD* mean difference

The effect of orthogeriatric care on TTS was examined in a meta-analysis. Out of the 19 studies that had data on TTS, 13 were included in the meta-analysis [21, 23, 25, 32, 34, 35, 39, 43–47, 51] (Fig. 3), while six were not because the data did not fulfill the requirements for the meta-analysis [22, 24, 30, 33, 38, 42].

The random-effects model showed a significant total mean difference of 0.23 days [95% CI (− 0.46, 0.01)], indicating that orthogeriatric care reduced TTS with 0.23 days (5.52 h) as compared to SOC. However, this result was not significant. In this analysis, three studies were identified as outliers: Biber et al., Duaso et al., and Gregersen et al. [35, 43, 46]. Therefore, these three studies were not retained in the result of the meta-analysis. The heterogeneity of the overall result is moderately high (*I*^2^ = 61%), which could complicate the interpretation of the results.

In the subgroups ICM, GCS, and GW, there was a significant reduction in TTS with 0.40 days (9.6 h) in the GCS subgroup, with 95% CI [− 0.52; − 0.28]. Heterogeneity was very low (*I*^2^ = 0%), indicating that all three studies showed similar results. The ICM subgroup showed a non-significant reduction in TTS with 0.29 days (6.96 h), with 95% CI [− 0.74; 0.16]. For the GW, only two studies were included, and the effect of this model was not significantly different from the control group.

#### In-hospital Mortality

**Fig. 4:**
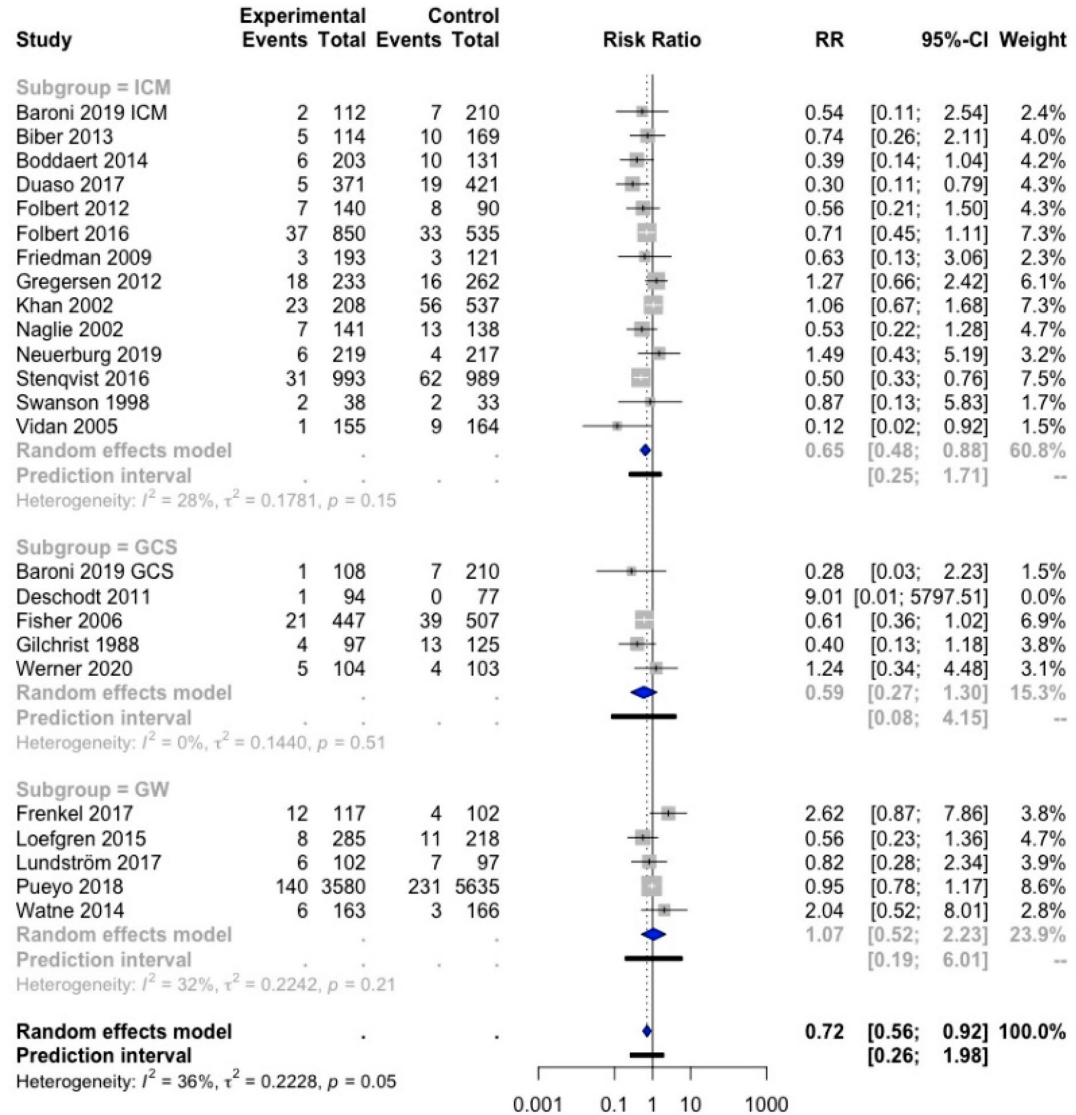
Forest plot of comparison of in-hospital mortality in hip fracture patients under orthogeriatric *vs.* usual orthopedic care. *ICM* integrated care model, *GCS* geriatric consultant service, *GW* geriatric ward, *RR* relative risk

Twenty-three studies provided data on in-hospital mortality and could be included in a meta-analysis [16, 20, 22–25, 27, 31–33, 35–38, 43–46, 48–52] (Fig. 4).

The combined result of the three orthogeriatric care models was significantly different from SOC, with a relative risk (RR) of 0.72; 95% CI [0.56; 0.92]. This indicates a 28% lower risk of in-hospital mortality for hip fracture patients included in an orthogeriatric care model as compared to SOC. No studies were outlying and heterogeneity was moderate (*I*^2^ = 36%). The study of Deschodt et al. [27] was removed from the result of the meta-analysis because RR was extremely high due to the fact the event of interest was not observed on one of the groups. Yet, this did not have a big impact on the results as the weight of this study was only 0.2%.

Subgroup analyses showed a significant result for the ICM subgroup, with a RR of 0.65; 95% CI [0.48; 0.88], indicating that hip fracture patients treated in an ICM care model are 35% less likely to die during hospital stay as compared to SOC. The results of GCS and GW were not significantly different from SOC.

#### 1-Year Mortality

**Fig. 5:**
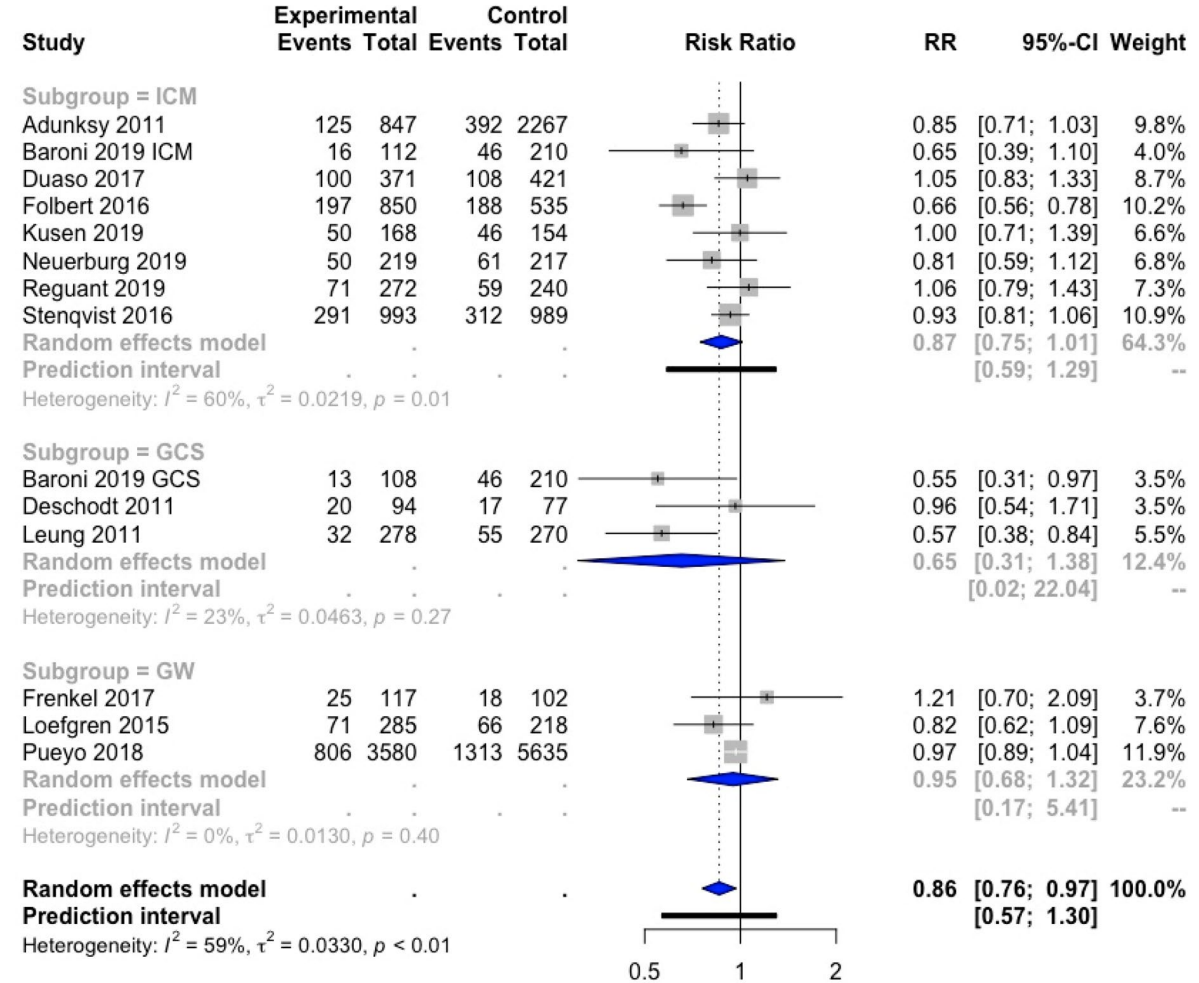
Forest plot of comparison of one-year mortality in hip fracture patients under orthogeriatric *vs.* usual orthopedic care. *ICM* integrated care model, *GCS* geriatric consultant service, *GW* geriatric ward, *RR* relative risk

All studies except Shyu 2008 et al. [18] that reported data on 1-year mortality could be included in a meta-analysis [27, 31, 32, 35, 38, 39, 41, 42, 44, 47–50] (Fig. 5). No studies were outlying.

The overall result was significant, with a relative rate of 0.86; 95% CI [0.78; 0.97], indicating that orthogeriatric care (all models combined) resulted in a 14% lower risk of 1-year mortality as compared to SOC. Subgroup analyses showed no significant differences in 1-year mortality between each orthogeriatric care model separately compared to SOC.

#### 30-day Readmission Rate

**Fig. 6:**
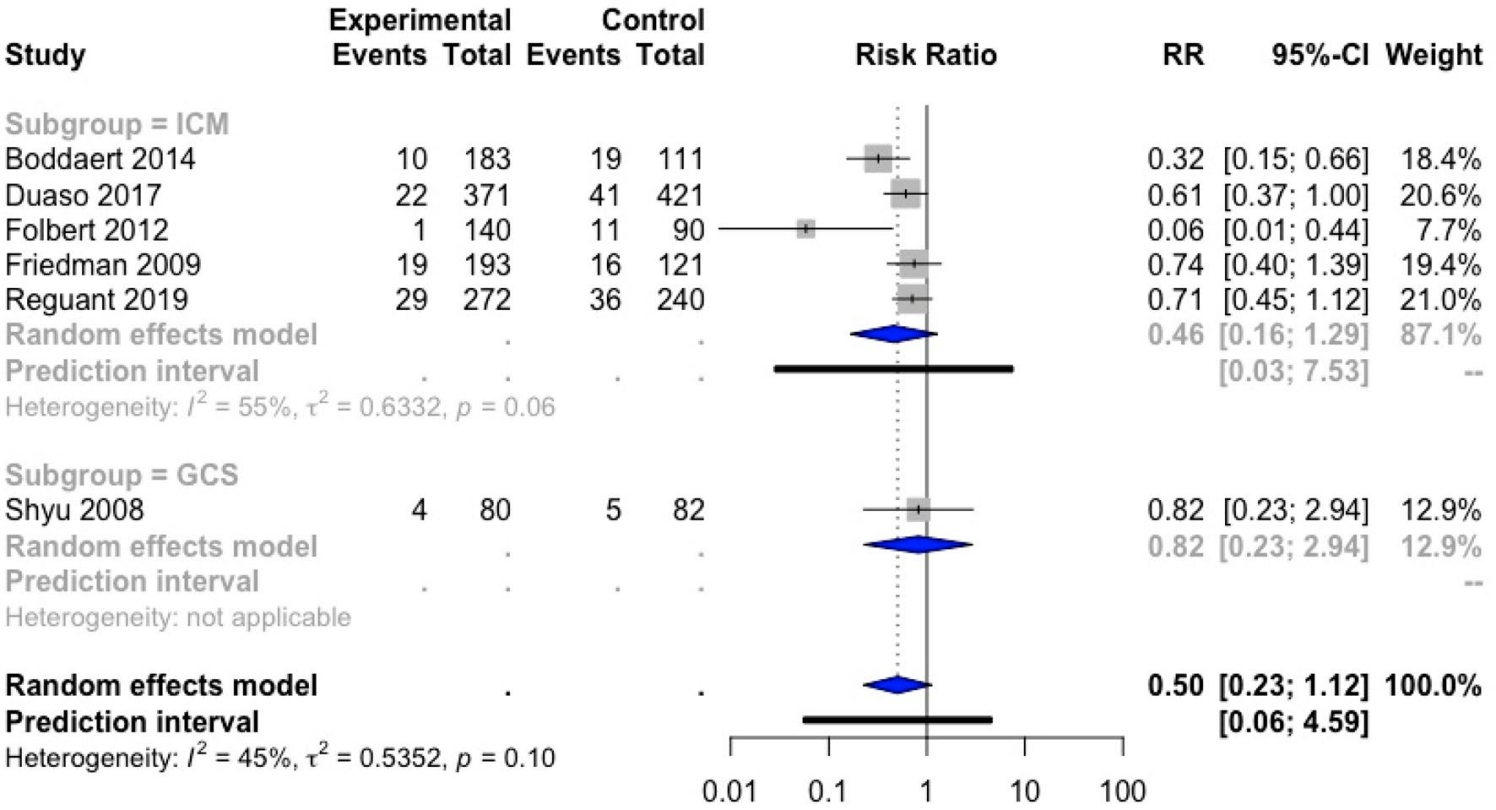
Forest plot of comparison of 30-day readmission rate in hip fracture patients under orthogeriatric *vs.* usual orthopedic care. *ICM* integrated care model, *GCS* geriatric consultant service, *GW* geriatric ward, *RR* relative risk

The impact of orthogeriatric care on 30-day readmission rate was evaluated in a meta-analysis of six studies [18, 33, 35, 37, 41, 45] (Fig. 6). No outliers were identified.

The overall effect was not significant [RR 0.50; 95% CI (0.23; 1.12)]. However, only a small amount of data was included in the meta-analysis. No conclusion could be drawn on the effect of the three subgroups separately because of the low number of trials included in each subgroup (ICM: five studies, GCS: one study, GW: no study).

#### Functional Outcome

Six articles reported data on functional outcome, evaluated by the performance of activities of daily living (ADL) (Table 3) [18, 21–23, 27, 34]. A meta-analysis could not be performed because of the heterogeneity of the trials regarding duration, follow-up, and the scale to assess functional outcome.

**Table 3:** Functional outcome

Study	ADL scale	Care model	FU (m)	ADL score of intervention group	ADL score of control group	*p* value
Bano et al. [34]	Katz index 0 = fully dependent 6 = fully independent	ICM	6	Mean loss (SD) 1.1 (1.7)	Mean loss (SD) 2.4 (2.2)	** < 0.001**
Deschodt et al. [27]	Katz index 6 = fully independent 18 = fully dependent	GCS	4 12	Mean (SD) 10.0 (3.8) 9.8 (3.8)	Mean (SD) 10.8 (3.9) 10.0 (3.4)	0.19 0.34
Prestmo et al. [21]	Barthel index 0 = fully dependent 20 = fully independent	GW	1 4 12	Mean (SE) 14.53 (0.28) 16.31 (0.29) 16.46 (0.29)	Mean (SE) 14.21 (0.29) 15.30 (0.29) 15.33 (0.30)	0.43 **0.013** **0.007**
Watne et al. [22]	Barthel index 0 = fully dependent 20 = fully independent	GW	4 12	Median (IQR) 17 (10–20) 17 (9.5–19)	Median (IQR) 16 (12–20) 16 (11–19)	0.80 0.44
Naglie et al. [23]	Modified Barthel index 0 = fully dependent 100 = fully independent	ICM	3 6	Mean (SD) 62.0 65.0	Mean (SD) 62.4 65.7	NS NS
Shyu et al. [18]	Chinese Barthel index 0 = fully dependent 100 = fully independent	GCS	1 3 6 12	Mean (SD) 81.24 (15.49) 88.82 (13.37) 91.84 (11.41) 90.53 (18.40)	Mean (SD) 72.92 (19.77) 79.93 (20.00) 84.08 (18.71) 84.36 (24.02)	*p* value for ADL performance trajectory: **0.002**

Bold values denote statistical significance at the *p* < 0.05 level

*ADL* activity of daily living, *GW* geriatric ward, *GCS* geriatric consultant service, *ICM* integrated care model, *FU* follow-up, *SD* standard deviation, *IQR* interquartile range, *SE* standard error, *NA* not assessed, *m* month

Bano et al. assessed functional outcome by using the Katz Index of Independence in Activities of Daily Living (ADL), which measures (in)dependence in six self-care activities (bathing, dressing, transfers, toileting, feeding, incontinence) [34]. Higher scores indicate higher independence (0 = fully dependent, 6 = fully independent). ADL score of hip fracture patients at 6 months after discharge was similar in the intervention group (ICM) and the control group (SOC), but in the ICM, a significantly lower functional decline (= loss in ADL score) was observed [34]. Deschodt et al. used an adapted version of the six-item Katz Index (1 = independent, 2 = partially dependent, 3 = dependent) with scores ranging from 6 to 18, where the higher scores indicate higher dependence (6 = fully independent, 18 = fully dependent) [27]. A higher independence was seen in the intervention group (GCS) at 4 and 12 months after hip fracture replacement, but these results were not significantly different from the control group (SOC) [27].

Watne et al. and Prestmo et al. evaluated functionality using the Barthel Index, which is a scoring system of the ability to perform ten ADLs (bathing, dressing, transfers, toilet use, feeding, bladder control, bowel control, climbing stairs, mobility, and grooming). The score ranges from 0 to 20 points, with a higher score for higher independence [21, 22]. In the trial by Watne et al., the difference in Barthel Index between the intervention group (GW) and SOC was not significant (neither at 4 months, nor at 12 months of follow-up) [22], neither was the result at 1 month in the trial by Prestmo et al. (GW *vs.* SOC) [21]. There was, however, in the trial by Prestmo et al., a significantly better Barthel Index in the intervention group (GW) at 4 months and 12 months as compared to SOC [21].

Naglie et al. assessed the modified Barthel index, which ranges from 0 (totally dependent) to 100 (totally independent), at 3 and 6 months of follow-up, and found no statistically significant difference between the intervention (ICM) and control group [23]. Finally, Shyu et al. evaluated the ability to perform ten ADLs, measured by the Chinese Barthel index (CBI), from 1 to 12 months after discharge [18]. The score of the CBI ranges from 0 (totally dependent) to 100 (totally independent). Compared to SOC, the intervention group (GCS) had a significantly better ADL trajectory during the first year after discharge.

To summarize, the effect of orthogeriatric care on functional outcome was inconsistent, with patients admitted to ICM as well as to GW and GCS showing better ADL performance (significantly reduced ADL loss after 6 months for ICM [34], better ADL performance after 4 and 12 months for GW [21] and better ADL performance trajectory over 12 months for GCS [18]) or no difference compared to SOC (at 3 and 6 months for ICM [23] and at 4 and 12 months for GW or GCS [22, 27]).

#### Complication Rates

Complications in hip fracture patients were evaluated in 18 trials (Table 4) [17, 20, 22, 25, 28, 32, 33, 35–39, 41, 43–45, 47, 51]. The data were very heterogeneous, with the evaluated complications varying largely, from total complications over different medical or surgical complications to delirium only.

**Table 4:** Complication rates

Studies	Care model	Type of complication	Intervention *N* (%) with complications	Control *N* (%) with complications	*p* value
Baroni et al. [32]	GCS	In-hospital complications^a^: ACS, arrhythmia, syncope, PE, stroke, MI, pneumonia, GI or other major bleeding, CHF, respiratory failure, acute renal failure, delirium, bed sore, UTI, DVT, wound infection, dysphagia, uncontrolled pain, vomiting, hypotension, electrolyte imbalance	99 (24.2%)	159 (38.8%)	NS
	ICM		85 (20.8%)	159 (38.8%)	** < 0.05**
Biber et al. [43]	ICM	Surgical complications: arthroplasty dislocation, hematoma or seroma, infection, other complication requiring revision surgery	9.6%	7.7%	0.6
Boddaert et al. [33]	ICM	Delirium Swallowing disorders Blood transfusion Stool impaction Urinary retention Pressure ulcer Acute heart failure Infection Venous thromboembolism Falls Admission to ICU	72 (35%) 56 (28%) 141 (69%) 83 (41%) 57 (28%) 18 (9%) 33 (16%) 40 (20%) 10 (5%) 9 (4%) 8 (4%)	49 (41%) 8 (7%) 72 (55%) 23 (19%) 26 (22%) 40 (33%) 6 (5%) 31 (25%) 1 (1%) 9 (7%) 17 (13%)	0.29 ** < 0.001** **0.008** ** < 0.001** 0.24 ** < 0.001** **0.002** 0.27 0.06 0.32 **0.005**
Deschodt et al. [28]	GCS	Postoperative delirium	35 (37.2%)	41 (53.2%)	**0.04**
Duaso et al. [35]	ICM	Anemia/transfusion Respiratory complication Cardiological complication Infectious complication	151 (40.7%) 15 (4.0%) 31 (8.4%) 13 (3.5%)	254 (60.3%) 29 (6.9%) 48 (11.4%) 32 (7.6%)	** < 0.001** 0.143 0.298 **0.027**
Fisher et al. [36]	GCS	Delirium Medical complications^a^: sepsis, delirium, pneumonia, venous thromboembolism, pressure sores, UTI, anemia, GI bleeding, ACS, CVA	5.9% 49.5%	11.7% 71.0%	**0.020** ** < 0.001**
Folbert et al. [37]	ICM	Delirium Medical & surgical complications^b^: UTI, urinary retention, wound infection, pneumonia, CHF, MI, osteosynthesis failure, renal failure, hypervolemia, electrolyte imbalance, anemia, nerve injury, pressure sore	54 (39%) Median (IQR) per patient: 0 (0–1)	30 (33%) Median (IQR) per patient: 1 (0–2)	0.421 **0.017**
Folbert et al. [38]	ICM	Medical & surgical complications^a^: -Medical: delirium, anemia, UTI, pneumonia, CHF, arrhythmia, renal failure, hypoxemia, MI, CVA, PE, other -Surgical: wound infection, dislocation implant, failure implant, re-intervention	454 (53.4%)	358 (66.9%)	** < 0.001**
Frenkel et al. [44]	GW	Delirium Medical complications^a^: UTI, urinary retention, acute renal failure, pneumonia, CHF, MI, CVA, delirium, PE, atrial fibrillation, SIRS, COPD exacerbation, other Orthopedic complications: wound infection, dislocation, per-prostatic fracture, reoperation	6 (5.2%) N (SD): 1 (1.2) per pt 12 (10.3)	5 (4.9%) N (SD): 0.6 (1) per pt 10 (9.8)	1.0 **0.029** 1.0
Friedman 2009 [45]	ICM	Delirium Medical & surgical complications^a^: renal failure, delirium, hypoxia, pneumonia, CHF, CVA, MI, surgical site infection, UTI, DVT, PE, hemorrhagic stroke, intracranial or retroperitoneal bleeding, GI bleeding, another fracture, implant dislocation, periprosthetic fracture, arrhythmia	24.4% 30.6%	32.2% 46.3%	0.13 **0.005**
Kusen et al. [39]	ICM	Delirium Medical & surgical complications^a^: -Medical: pneumonia, delirium, UTI, CHF, decubital ulcer, CVA, PE, renal insufficiency, reanimation, GI bleeding -Surgical: wound infection, hematoma, anemia, loss of reduction, screw cut-out/through, nail breakage, loss of fixation, joint infection	22 (13.1%) 89 (53.0%)	3 (1.9%) 85 (55.2%)	** < 0.001** 0.69
Leung et al. [47]	GCS	Postoperative complications	154 (55.4%)	155 (57.4%)	0.54
Lundström et al. [20]	GW	Anemia Constipation Pressure ulcer Delirium Heart failure Pneumonia Urinary infection Myocardial infarction Nutritional complications Pulmonary embolism Stroke Urinary retention Falls	88 (86.3%) 38 (37.3%) 9 (8.8%) 56 (54.9%) 6 (5.9%) 5 (4.9%) 32 (31.4%) 2 (2.0%) 25 (24.5%) 2 (2.0%) 0 16 (15.7%) 12 (11.8%)	79 (82.3%) 47 (48.5%) 21 (22.1%) 73 (75.3%) 11 (11.6%) 3 (3.1%) 49 (51.0%) 4 (4.1%) 37 (38.1%) 0 1 (1.0%) 18 (18.6%) 26 (26.8%)	0.441 0.110 **0.010** **0.03** 0.161 0.772 **0.005** 0.436 0.038 0.498 0.485 0.591 **0.006**
Marcantonio et al. [17]	GCS	Delirium	20 (32%)	32 (50%)	**0.04**
Reguant et al. [41]	ICM	Delirium Medical & surgical complications^a^: cardiovascular, respiratory, secondary to spinal anesthesia, severe bleeding, renal, infections, re-intervention, cognitive disorders	62 (22.8%) 183 (67.3%)	66 (27.5%) 183 (76.2%)	0.220 **0.025**
Vidan et al. [25]	ICM	Delirium Medical complications^a^: delirium, CHF, pneumonia, DVT, PE, pressure ulcers, arrhythmia, MI	53 (34.2%) 70 (45.2%)	67 (44.1%) 100 (61.7%)	0.07 **0.003**
Watne et al. [22]	GW	Delirium Medical complications^b^: cardiac, cerebral, thrombo-embolic, pulmonary, GI, renal failure, UTI, pressure ulcer Surgical complications: surgical site infection, wound problem, osteosynthesis failure, dislocation of prosthesis	80 (49%) 72 (44%) 4 (3%)	86 (53%) 76 (46%) 6 (4%)	0.51 0.82 0.75
Werner et al. [51]	GCS	Delirium Medical & surgical complications^b^ pressure sores, UTI, acute kidney injury, GI bleeding, ileus, pneumonia, MI, PE, DVT, CVA, implant failure or luxation, wound infection	41 (39.4%) 26 (25.0%)	42 (40.8%) 25 (24.3%)	0.888 > 0.99

Bold values denote statistical significance at the *p* < 0.05 level

*GW* geriatric ward, *GCS* geriatric consultant service, *ICM* integrated care model, ^a^including delirium, ^b^excluding delirium, *ACS* acute coronary syndrome, *CVA* cerebrovascular accident, *CHF* congestive heart failure, *DVT* deep venous thrombosis, *GI* gastrointestinal, *MI* myocardial infarction, *pt* patient, *PE* pulmonary embolism, *SIRS* systemic inflammatory response syndrome, *UTI* urinary tract infection

In general, most complications occurred numerically less frequent in the intervention groups (orthogeriatric care) than in the control groups (SOC), and for medical and/or surgical complications as a whole, statistical significance for lower complications in the intervention groups was reached in Baroni et al. [32], Fisher et al. [36], Folbert 2012 and 2017 et al. [37, 38], Friedman et al. [45], Reguant et al. [41], and Vidan et al. [25]. With respect to specific complications, some evidence was found for a significantly lower incidence of pressure ulcers [20, 33], anemia/transfusion [35], urinary tract infections [20], and falls [20] in orthogeriatric care groups. On the contrary, Frenkel et al. observed significantly more medical complications in the intervention group (GW) [44]. Likewise, certain events such as obstipation, swallowing disorders, transfusions, and acute heart failure were reported significantly more often in the intervention group (ICM) in Boddaert et al. [33].

Looking at the orthogeriatric care models separately, a significant increase in medical complications was observed in the GW in the study of Frenkel et al. [44], but a significant lower incidence of complications (more specifically delirium, pressure ulcers, urinary tract infections, and falls) was observed in the GW in Lundström et al. [20]. From the six trials studying the effect of GCS, three showed significantly lower complication rates for GCS as compared to SOC. More specifically, a lower incidence of medical complications [36] and delirium [17, 28, 36] was observed. Other GCS studies, however, only found insignificant differences between GCS and SOC [32, 47, 51]. For the ICM, eight of the ten trials showed significant reductions in at least some of the assessed complications [25, 32, 33, 35, 37, 38, 41, 45], and a significant higher incidence of obstipation, swallowing disorders, transfusions, and acute heart failure was observed in the ICM in Boddaert et al. [33]. Finally, Baroni et al. observed a significantly lower number of in-hospital complications in ICM *vs*. SOC, but not in GCS *vs.* SOC [32].

**Fig. 7:**
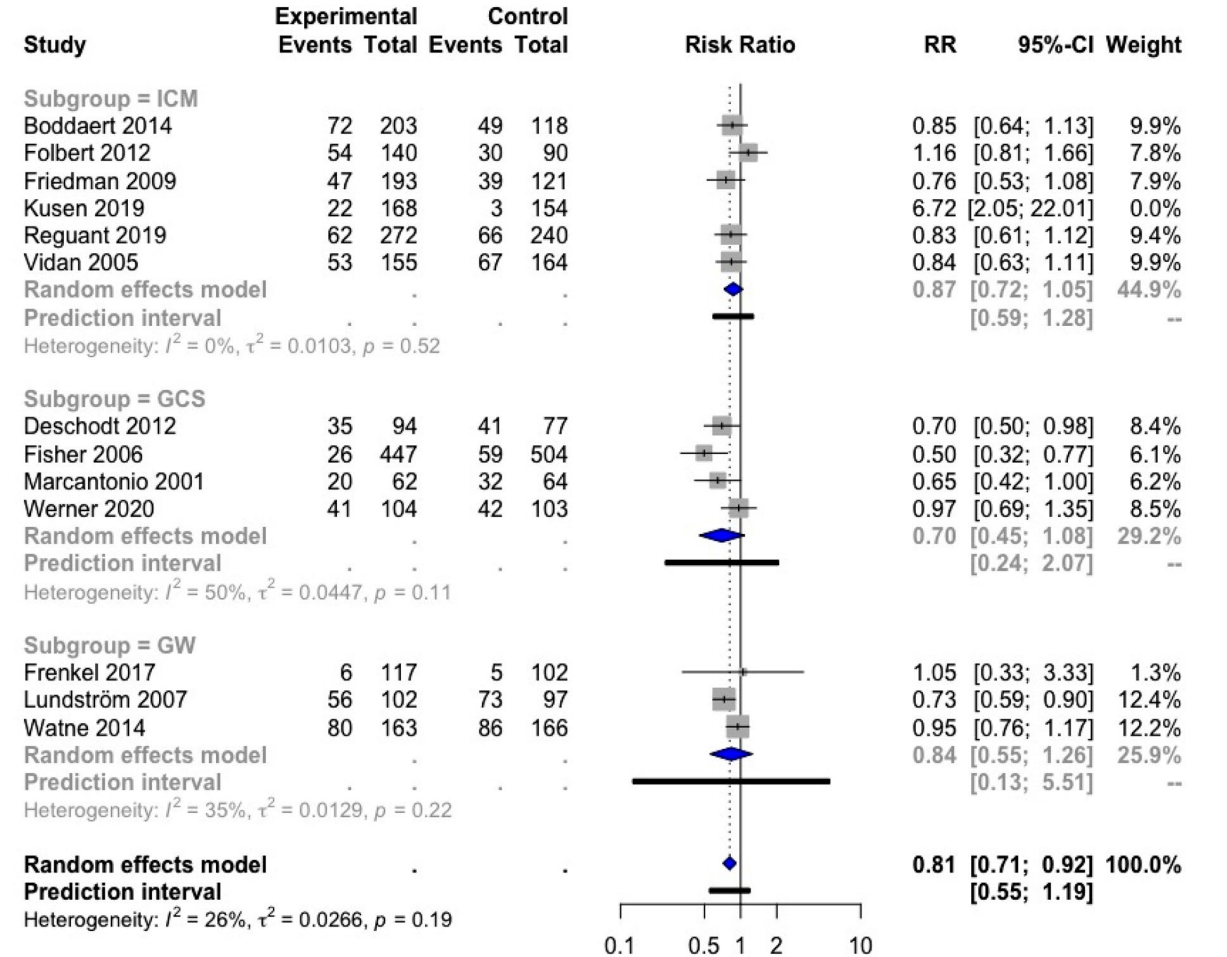
Forest plot of comparison of delirium in hip fracture patients under orthogeriatric *vs.* usual orthopedic care. *ICM* integrated care model, *GCS* geriatric consultant service, *GW* geriatric ward, *RR* relative risk

We found 15 studies reporting results on delirium incidence; 13 of these could be included in a meta-analysis: six in ICM [25, 33, 37, 39, 41, 45], four in GCS [17, 28, 36, 51], and three in GW [20, 22, 44]. The study of Kusen et al. [39] was considered an outlier based on the influence analysis and funnel plot and was therefore excluded. The overall result of this meta-analysis on delirium was significant, with a RR of 0.81; 95% CI [0.71; 0.92] (Fig. 7). The heterogeneity was low, only 26%, indicating a rather low variability between the different trials. The subgroup analyses were all in favor of the respective orthogeriatric care model, but these results were not significant.

#### Cost Associated with Orthogeriatric Care Models

Five studies examined the costs associated with orthogeriatric care models [21, 26, 29, 31, 40] (Table 5). In some of these studies, only the cost for the inpatient care episode was included (with or without revalidation) [31, 40], while other studies included all costs during a 12-month or even an 18-month follow-up period [21, 26, 29]. In the latter case, calculated costs may also include, apart from the medical and allied health follow-up, management of secondary fractures [26]. In all included studies, mean total cost per patient was numerically lower in the intervention group than in the control group.

**Table 5:** Cost associated with orthogeriatric care models

Studies	Care model	Included costs	Value	Cost in intervention group, mean (SD)	Cost in control group, mean (SD)	*p* value
Cheung et al. [26]	GCS	Total cost per patient during 18-month follow-up	US $	22.450	25.313	NA
Löfgren et al. [31]	GW	Cost per patient for whole care episode	SEK	115.163	124.879	NA
Prestmo et al. [21]	GW	Total cost per patient during 12-month follow-up	Euro	54.332 (38.048)	59.486 (44.301)	0.22
Miura et al. [40]	GW	Direct and indirect cost per patient in inpatient period	US $	9.109 (2.326)	11.299 (4.808)	** < 0.001**
Ginsberg et al. [29]	ICM	Total cost per patient during 12-month follow-up	US $	14.919	19.363	NA

One SEK equals 0.009 Euro. One US $ equals 0.83 Euro

Bold values denote statistical significance at the *p* < 0.05 level

*GW* geriatric ward, *GCS* geriatric consultant service, *ICM* integrated care model, *NA* not assessed, *SD* standard deviation, *SEK* Swedish Krona, *US $* United States dollar

Prestmo et al. also evaluated the number of quality-adjusted life years (QALYs) at 4 and 12 months, which was higher in orthogeriatric care (GW) than in SOC [21]. In addition, Ginsberg et al. showed that orthogeriatrics (ICM) did not only use 23% fewer resources per patient, but also increased disability-adjusted life years (DALYs) with 0.226 per patient [29]. These data suggest that orthogeriatric care models are a cost-effective alternative to SOC.

## Discussion

The aim of this systematic review and meta-analysis was to investigate whether orthogeriatric care models for hip fracture patients improve LOS, TTS, in-hospital mortality, 1-year mortality, 30-day readmission rate, functional outcome, complication rate, and cost.

With a meta-analysis, we found evidence that orthogeriatric care (three models combined) for hip fracture patients significantly reduced LOS (− 1.55 days), but not TTS. In the subgroups (each model separately), heterogeneity was moderate (LOS) to low (TTS) in GCS only, showing a significant reduction of LOS by 1.37 days and a significant reduction of TTS by 0.40 days. Another meta-analysis showed, with moderate heterogeneity, that orthogeriatric care also resulted in a lower risk of in-hospital (RRR 28%) and 1-year mortality (RRR 14%), but in the subgroup meta-analyses, only the ICM was found to result in a significant lower risk of in-hospital mortality (RRR 35%). An impact on 30-day readmission rate was not observed, but only a limited number of studies was included in this meta-analysis. The data for functional outcome, complication rate, and costs were difficult to interpret because of substantial heterogeneity. No consistent effect of orthogeriatric care was found on functional outcome. In general, numerically lower numbers of complication rates were observed in orthogeriatric care, although some complications, including delirium and other geriatric syndromes, were observed more frequently in some of the GWs and ICMs. A meta-analysis on the effect on delirium showed a significant reduction of delirium in orthogeriatric care as a whole, but the subgroup analyses were not significant. Limited data suggest that orthogeriatrics could be cost-effective. The evidence was insufficient to compare different care models directly against each other. Table 6 summarized the results of the meta-analyses of LOS, TTS, in-hospital mortality, 1-year mortality, 30-day readmission rate, and delirium.

**Table 6:** Summary meta-analysis results

	Length of stay	Time to surgery	In-hospital mortality	1-year mortality	30-day readmission rate	delirium
	MD; 95% CI *I*^2^, *p* *n*	MD; 95% CI *I*^2^, *p* *n*	RR; 95% CI *I*^2^, *p* *n*	RR; 95% CI *I*^2^, *p* *n*	RR; 95% CI *I*^2^, *p* *n*	RR; 95% CI *I*^2^, *p* *n*
3 Models	**1.55; 95% CI [− 2.53; -0.57]**	− 0.23; 95% CI [− 0.46; 0.01]	**0.72; 95% CI [0.56; 0.92]**	**0.86; 95% CI [0.76; 0.97]**	0.50; 95% CI [0.23; 1.12]	**0.81; 95% CI [0.71; 0.92]**
	80%, *p* < 0.01	61%, *p* < 0.01	36%, *p* = 0.05	59%, *p* < 0.01	45%, *p* = 0.10	26%, *p* = 0.19
	*N* = 24^a,b^	*N* = 13^b,c^	*N* = 23^b^	*N* = 13^b^	*N* = 6	*N* = 13^d^
ICM	**− 2.12; 95% CI [−4.22; -0.03]**	− 0.29; 95% CI [− 0.74; 0.16]	**0.65; 95% CI [0.48; 0.88]**	0.87; 95% CI [0.75; 1.01]	0.46; 95% CI [0.16; 1.29]	0.87; 95% CI [0.72; 1.05]
	81%, *p* < 0.01	69%, *p* < 0.01	28%, *p* = 0.15	60%, *p* = 0.01	55%, *p* = 0.06	0%, *p* = 0.52
	*N* = 11^a^	*N* = 9^c^	*N* = 14	*N* = 8	*N* = 5	*N* = 6^d^
GCS	**− 1.37; 95% CI [− 2.42; − 0.33]**	**− 0.40; 95% CI [− 0.52; − 0.28]**	0.59; 95% CI [0.27; 1.30]	0.65; 95% CI [0.31; 1.38]	0.82; 95% CI [0.23; 2.94]	0.70; 95% CI [0.45; 1.08]
	61%, *p* = 0.01	0%, *p* = 0.94	0%, *p* = 0.51	23%, *p* = 0.27	NA	50%, *p* = 0.11
	*N* = 8	*N* = 3	*N* = 5	*N* = 3	*N* = 1	*N* = 4
GW	− 1.24; 95% CI [− 4.85; 2.37]	0.05; 95% CI [-1.42; 1.51]	1.07; 95% CI [0.52; 2.23]	0.95; 95% CI [0.68; 1.32]	NA	0.84; 95% CI [0.55; 1.26]
	88%, *p* < 0.01	16%, *p* = 0.27	32%, *p* = 0.21	*p*0%, = 0.40	NA	35%, *p* = 0.22
	*N* = 6	*N* = 2	*N* = 5	*N* = 3	*N* = 0	*N* = 3

Bold values denote statistical significance at the *p* < 0.05 level

*ICM* integrated care model, *GCS* geriatric consultant service, *GW* geriatric ward, *MD* mean difference, *RR* relative risk, *CI* confidence interval, *I*^2^ test for heterogeneity, *p* significance for heterogeneity, *n* number of included studies in meta-analysis, *NA* not applicable

^a^Duaso et al. [35] and Naglie et al. [23] are excluded from the result of the meta-analysis because these studies were outlying on the overall LOS result

^b^Baroni et al. [32] investigated both ICM and GCS

^c^Biber et al. [43], Duaso et al. [35], and Gregersen et al. [46] are excluded from the result of the meta-analysis because these studies were outlying on the overall TTS result

^d^Kusen et al. [39] was excluded from the result of the meta-analysis because the study was outlying on the overall delirium result

### Length of Stay

In the individual trials that evaluated the effect of orthogeriatric care for hip fracture patients on LOS, we observed both decreases and increases in LOS. This is explained by the fact that, although orthogeriatric care with comprehensive geriatric assessment (CGA) and extensive discharge planning may be time-consuming [21, 44], early and coordinated discharge planning and possibly also shorter TTS, lower complication rates, and timely management of complications associated with orthogeriatric care may result in a shorter LOS [35].

With a meta-analysis, we showed that orthogeriatric care for hip fracture patients, compared to SOC, significantly reduced LOS with 1.55 days. More specifically, ICM and GCS reduced LOS by 2.12 and 1.37 days, respectively, while GW had no significant effect on LOS. Importantly, the findings from the meta-analysis that combines the three orthogeriatric care models as well as the meta-analyses of ICM and GW separately should be taken with caution because of large heterogeneity. Part of this heterogeneity is explained by the great variability of LOS among included studies, which ranges from 4.6 days [45] to 47.7 days [16]. Among other, LOS depends on the local organization of the healthcare system (e.g., whether or not quick discharge to a nursing home is possible) and on the modalities of the care program (e.g., whether or not the rehabilitation phase is included in the hospital stay). To illustrate, the high LOS in Gilchrist et al. is explained by the fact that patients who went home shortly after surgery were excluded and only those who were transferred for rehabilitation in GCS or SOC were included in the analysis. This may have contributed to the fact that GCS resulted in a 3.7-day shorter LOS compared to SOC, as the patients who were not discharged early (and thus could be included in the trial) may have had a typically geriatric profile and, therefore, may have benefited more from the orthogeriatric care [16]. In contrast, the ability to transfer patients to rehabilitation units without delay contributed to the short LOS in the ICM group of Duaso et al., which is significantly shorter (63%) than in SOC [35]. It should be noted that we excluded the study of Duaso from both the combined and the ICM subgroup meta-analyses because the study results were outlying and highly influenced the results of these meta-analyses.

An earlier meta-analysis on orthogeriatric care for hip fracture patients, by Grigoryan et al., also found that orthogeriatrics was associated with a significantly reduced LOS [SMD − 0.25; 95% CI (− 0.44; − 0.05)] [11]. However, in particular in the ICM model [SMD − 0.61; 95% CI (− 0.95; − 0.28)] and not in the GCS model [SMD − 0.03; 95% CI (− 0.20; 0.14)] as we observed in our meta-analysis. In this earlier meta-analysis as well, large heterogeneity limits the possibility to draw firm conclusions [11].

A short(er) LOS is used as an indicator of the efficacy of the provided care. In addition, it may be associated with a lower total cost per patient. However, since aforementioned factors such as healthcare organization contribute to LOS, the percentage of patients discharged within a specified time frame may be a more useful quality indicator than LOS, as suggested by Baroni et al. [32]. In addition, from a geriatric perspective, a slightly longer LOS may be acceptable, when this is associated with a greater functional recovery at discharge or a higher chance of being discharged directly home [21, 24].

### Time to Surgery

The overall meta-analysis did not show a significant effect of orthogeriatric care on TTS. However, in the GCS subgroup alone, TTS was significantly reduced by 0.40 days, with very low heterogeneity between the three included studies. The effect in the GW subgroup is difficult to interpret because of the limited number of trials in this meta-analysis. These results are in agreement with the earlier meta-analysis of Grigoryan et al., that showed a significant decrease in TTS for hip fracture patients admitted to GCS [SMD − 0.13; 95% CI (− 0.23; − 0.03); *I*^2^ 0.0%], but large heterogeneity in the ICM subgroup and too few studies to perform a GW meta-analysis [11]. Interestingly, the study of Baroni et al. is the only one that directly compared the effect of two orthogeriatric care models on TTS and found that ICM was superior to GCS in shortening TTS [32].

TTS is an important parameter in the care of hip fracture patients because of its association with mortality. This is explained by the increased risk of respiratory, cardiovascular, thrombotic, and infectious complications resulting from confinement to bed and preoperative stress when surgery is delayed [53]. A recent meta-analysis of 28 prospective observational studies reporting data of 31.242 patients showed that hip fracture patients operated on within 48 h had a 20% lower risk of dying within 12 months [RR 0.80: 95% CI (0.66; 0.97)], while no statistical significant difference in mortality risk was observed when comparing patients operated on within or after 24 h [RR 0.82; 95% CI (0.67; 1.01)] [54]. In the individual studies included in our meta-analyses, TTS varied from 0.99 [39] to 4.50 [32] days in SOC and from 0.82 [39] to 4.20 [32] days in orthogeriatric care. TTS was > 48 h in six of the 14 control groups (SOC) [25, 26, 32, 35, 43, 47] and in three of the 14 intervention groups (ICM or GCS) [25, 32, 43]. Remarkably, in one study TTS significantly increased in the ICM subgroup as compared to SOC [46]. Delay in surgery may be explained by patient-related factors such as medical instability and the need to treat comorbidities, but hospital-related factors such as limited capacity of operating rooms and qualified personnel, for example during weekends, explain up to 75% of the delays in surgery [54].

Thus, shorter TTS, more specifically a period less than 48 h between hip fracture diagnosis and surgery, may be associated with lower mortality risk. However, ultra-early surgery (within 6 h) as compared to SOC [median time to surgery 24 h (IQR 10–42)] in an RCT with 2970 hip fracture patients aged ≥ 45 years did not reduce the risk of mortality nor the composite endpoint of major complications (i.e., mortality and non-fatal myocardial infarction, stroke, venous thromboembolism, sepsis, pneumonia, life-threatening bleeding, and major bleeding) at 90 days after randomization [55]. However, a lower risk of delirium and urinary tract infections as well as faster standing, mobilization, and hospital discharge was observed. Ultra-accelerated hip fracture surgery may negatively impact major postoperative outcomes, including survival, by limiting the opportunity to optimize the medical conditions of a patient before surgery. In none of the studies included in our meta-analyses, TTS approached 6 h.

### In-hospital and 1-year Mortality

Hip fracture patients admitted to orthogeriatric care had a 28% lower risk of in-hospital mortality and a 14% lower risk of 1-year mortality as compared to those admitted to SOC. For the subgroup analyses, only in the ICM subgroup, the risk of in-hospital mortality was significantly decreased. A trend to lower in-hospital mortality and a trend to lower 1-year mortality were observed for the GCS and GW subgroups, and for the ICM, GCS, and GW subgroups, respectively.

Our data partly confirm the results of previous meta-analyses [11, 12]. Grigoryan et al. showed that orthogeriatric collaboration as a whole significantly reduced in-hospital mortality by 40% [RR 0.60; 95% (0.43; 0.84)—nine studies] and long-term mortality by 17% [RR 0.83; 95% CI (0.74; 0.94)—eleven studies]. Long-term mortality was defined as mortality from 6 months to 1 year. Looking at the individual models in Grigoryan et al., only the meta-analysis of the GCS model found a significant decrease in both in-hospital and long-term mortality. For GW and ICM, a low number of published trials and/or large heterogeneity reduced the ability to perform a meta-analysis and/or to draw final conclusions [11]. The more recent meta-analysis of Moyet confirmed the effect of orthogeriatric care on long-term mortality [OR 0.79; 95% CI (0.68; 0.93)—ten studies], but an effect on short-term mortality (in-hospital upon 3-month mortality) was not significant [OR 0.94; 95% CI (0.75; 1.18)—13 studies]. With respect to the subgroup analyses, mortality was significantly lower compared to SOC only in the GW subgroup [12]. So, our analyses confirm previous observations that orthogeriatric care improves (in-hospital and 1-year) mortality of hip fracture patients. While we found evidence that, more specifically, ICM results in reduced in-hospital mortality, other meta-analyses found reduced mortality for GW or GCS.

Mortality after hip fracture repair is determined by a number of factors. A recent prospective cohort study in 1083 hip fracture patients aged ≥ 65 years identified advancing age, higher baseline Charlson comorbidity index (CCI), higher pre-fracture dependence in ADL, hospital-acquired pressure ulcers, and lack of recovery from ambulation as independent predictors of 1-year mortality [56]. TTS, however, did not predict 1-year mortality, which can be explained by the fact that almost 80% of the included patients underwent surgery within 48 h after the fracture. By consequence, early surgery, early ambulation, and a CGA to identify frail patients and to treat them according to their needs are among the key elements of orthogeriatric care, in addition to prevention and management of delirium, pain, and malnutrition.

### 30-day Readmission Rate

The effect of orthogeriatric care as a whole on 30-day readmission rate was not significant. No conclusion could be made about the subgroups because of the limited number of included studies.

In a prospective study of 236 hip fracture patients, 30.1% was readmitted within 12 months of discharge and 41% of all readmissions occurred in the first 3 months after discharge. Surgical complicates accounted for less than 10% of the readmissions, and heart failure, stroke, and pneumonia together for 45% [57]. A systematic review on this topic confirmed a median 30-day readmission rate of 10.1%, with medical causes of readmission being up to 13 times more common than surgical causes. The ASA score (American Society of Anesthesiologists physical status classification system; a scoring system to assess the fitness of a patient before surgery) and functional status are more robust predictors of readmission than the CCI or individual comorbidities [58].

LOS, mortality, and 30-day readmission rate are closely related, with both LOS and readmission rate being strong predictors of mortality. While it is reassuring that reduced LOS does not necessarily increase readmission rate [59], prolonged LOS may be associated with both an increase and a decrease in the readmission rate (e.g., when the prolonged LOS is the result of medical problems or an extensive discharge planning, respectively) [58]. In addition to LOS and mortality, 30-day readmission rate is suggested as a metric of hospital performance. However, while LOS and mortality are predicted by hospital-related factors (e.g., type of anesthesia and TTS), 30-day readmission rate is predicted by patients-related factors such as age, comorbidity, functional status, and discharge location. This makes 30-day readmission rate a less valid metric of hospital performance [58].

More research is needed to determine the effect of orthogeriatric as a whole and of the different orthogeriatric care models on 30-day readmission rate.

### Functional Outcome

We found that the effect of orthogeriatrics on functional outcome (measured as ADL performance) was inconsistent, with patients admitted to ICM as well as to GW and GCS showing better ADL performance [18, 21, 34] or no difference compared to SOC [22, 23, 27].

We did not do a meta-analysis because of the heterogeneity in follow-up and ADL scale. Mukherjee et al., however, meta-analyzed the two trials [21, 22] that evaluated ADL by the Barthel index at 4 and 12 months in GW and SOC. A significant benefit for the GW was found at 4 months [MD 1.01; 95% CI (0.28; 1.73), *I*^2^ = 0%] that persisted at twelve months [MD 1.11; 95% CI (0.36; 1.85), *I*^2^ = 0%] [13].

In contrast to LOS, TTS, and (in-hospital and 1-year) mortality, a limited number of studies has evaluated the effect of orthogeriatric care on functional outcome. Yet, the main purpose of hip fracture repair is to restore the pre-fracture level of functional performance of hip fracture patients. Therefore, more RCTs with standardized orthogeriatric care protocols and outcome measures should be done to determine the effect of orthogeriatric care and its different modalities on functional outcome. In this context, it should be noted that the assessment of functional outcome by tools such as the Barthel index, although commonly used in clinical practice and sensitive to detect declines in health status, may be less sensitive to evaluate small changes in the functional performance of hip fracture patients [34]. Therefore, scales that are more sensitive to such subtle changes should be preferred, e.g., the modified Barthel index used by Naglie et al. [23], although in the end, a full CGA may be the most informative on the functional status of a geriatric patient.

### Complication Rates

In general, numerically less medical and surgical complications were observed in the intervention groups (orthogeriatric care) compared to SOC. This is explained by the introduction of the CGA (the process to assess a geriatric patient and to develop an individualized plan of treatment and follow-up in association with a multidisciplinary team) and the use of standardized protocols (e.g., for early mobilization and transfusion) in orthogeriatric care. Remarkably, we observed that some complications occurred more frequently in orthogeriatric care models; more specifically in the GW and the ICM, such as more medical complications in Frenkel et al. (GW) [44] and a higher incidence of obstipation, swallowing disorders, and acute heart failure in Boddaert et al. (ICM) [33]. This observation may, at least partly, be explained by the retrospective recording of the data in the SOC groups, but it is not excluded that these complications were detected more frequently in the orthogeriatric care models due to improved surveillance for typical geriatric syndromes such as obstipation and swallowing disorders [33].

We were able to perform a meta-analysis on the incidence of delirium and concluded that delirium occurred less frequently in orthogeriatric care settings. The study of Kusen et al. [39], however, reported an increase in delirium in the ICM group, which the authors attributed to an increased awareness, routine screening, and adequate registration of delirium in the orthogeriatric care group. In our meta-analysis on delirium, this study was considered an outlier and was therefore excluded. The significantly lower incidence of delirium that we observed in our meta-analysis could be explained by immediate and systematical screening for and adequate treatment and prevention of delirium in the groups with orthogeriatric care. Nevertheless, this result should be interpreted with caution since 7 from the 12 studies included in this meta-analysis are retrospective. Thus, information bias might play a role in the reported results. The level of awareness for delirium incidence depending on the setting (geriatric medical ward *vs.* orthopedic ward) might have influenced the reported incidence, although none of the subgroup analyses showed a significant result. Similar to our findings, the meta-analysis of Wang et al. on the effect of orthogeriatric care found a significant reduction in the incidence of perioperative delirium [OR = 0.71; 95% CI (0.57; 0.89); *p* = 0.03, *I*^2^ = 25%] [60].

Mukherjee et al. recently performed a meta-analysis on the effect of orthogeriatric care on hospital-acquired pressure ulcers and on medical complications. Compared to SOC, the orthogeriatric care group showed a lower risk of pressure ulcers [OR 0.30; 95% CI (0.15; 0.60), *I*^2^ = 0%], but not of medical complications [OR 0.70; 95% CI (0.40, 1.24), *I*^2^ = 70%]. The large heterogeneity in this meta-analysis of Mukherjee et al. and the fact that only two trials were included strongly limit the possibility to draw conclusions based on those data.

Further research is needed to determine which of the three orthogeriatric care models is the best to reduce complications. The study of Baroni et al. is the only one that directly compares two models: the number of in-hospital complications was significantly lower in ICM *vs.* SOC, and not in GCS *vs.* SOC, but there was no significant difference in the complication rate between ICM and GCS [32].

### Costs

A comparison between studies of the healthcare expenditure of orthogeriatric care is difficult because of the variations in follow-up (e.g., follow-up of 12 or 18 months) and costs included in the analyses (e.g., management of secondary fracture). In addition, also differences in guidelines, reimbursement criteria, healthcare budget, etc., between regional and national healthcare centers make a comparison of cost-effectiveness a complex exercise [31]. In general, we found that orthogeriatric care was associated with reduced cost. This may be explained by shorter LOS, lower complication rate, less out-patient department consultations, and better rehabilitation with lower risk of falls and refractures (although in the recent meta-analysis of Van Camp et al. the evidence of orthogeriatric care on fall prevention and subsequent fractures was scarce and inconclusive [61]). In combination with the effects on morbidity and mortality, orthogeriatric care is considered to be cost-effective [21, 29]. This supports the recommendation to implement orthogeriatric care for hip fracture patients in clinical practice [62].

### Demography

In Table 1, the countries of origin of the included studies were indicated. Out of 37 included articles, the majority (25) originated from European countries suggesting that orthogeriatric care models as evaluated in the present analysis are more established in Europe compared to other continents. The other 12 included articles originated from different geographical areas: Asia (three), USA (four), Australia (two), and the Middle-East (three). Thus, research related to orthogeriatric models may be more scarce outside Europe, which may be explained by the implementation of other types of care for hip fracture patients such as fracture liaison services or by the poor implementation of orthogeriatric care [63]. For example, the 2018 report of the Australia & New Zealand Hip Fracture Registry found that 55% of hospitals reported an orthogeriatric service for older hip fracture patients [64], while a cross-sectional survey in eight European countries reported some kind of orthogeriatric care (mainly geriatric consultation teams) in up to 100% of hospitals in Belgium and in more than 70% of hospitals in Ireland [65]. Also in Asian countries orthogeriatric care has not been routinely implemented [63, 66]. In addition, in some countries, such as in India and Japan, care for older people is not delivered by geriatricians, but by general internists [63, 67]. Studies that examined their collaboration with the orthopedic surgeon were not included in our study. Finally, we only included studies published in English, Dutch and French. Possibly, data from areas of the ‘geographical gap’ were published in other languages (e.g., Chinese, Russian, Spanish, etc.) and might have been missed for inclusion.

### Strengths and Limitations

This systematic review and meta-analysis have several strengths. One of the strengths is the large number of hip fracture outcomes that is evaluated. In addition, a large number of studies is included, some of which have not been included in a systematic review before. 30-day readmission rate is included in a meta-analysis for the first time, although the number of included studies turned out to be too low to make an overall conclusion on the effect of orthogeriatric care on this outcome.

Several limitations need to be taken into account as well. First, some studies showed high heterogeneity, were considered outlying or had too important influence on the overall result, making it difficult to interpret the data and to draw final conclusions. Second, not all studies could be included in the meta-analyses because of missing data although the authors were contacted to provide additional information. This could possibly bias some of the results. Other studies were lacking a control group or did not clearly describe the orthogeriatric intervention. Third, to assess the effect on some outcomes, especially of the separate orthogeriatric models, the number of studies that could be included was too low. Fourth, we did not perform an individual participant data meta-analysis. Another limitation is that the subdivision of the different care models is quite arbitrary. We made this distinction based on the involvement of an orthopedic surgeon and geriatrician as well as on the place where the care was given. However, in clinical practice, true orthogeriatric care additionally includes the expertise of a whole multidisciplinary team, including a nurse, physiotherapist, occupational therapist, dietician, speech therapist, and social worker. Fifth, through the title and abstract screening, 14 articles were identified that might have been suitable for the analyses in this study, but were excluded due to the lack of available full-text. The inclusion of these articles could have altered some of the results. Finally, although we included a large set of outcome parameters, we did not assess al the outcomes that were recommended by Liem et al. for the evaluation of orthogeriatric care of hip fracture patients [68]. In addition to LOS, TTS, mortality, readmission rate, ADL, postoperative complications, and cost, Liem et al. suggested to also evaluate mobility, quality of life, pain, medication use and place of residence, and so at admission and discharge, and at 30 days, 90 days, and 1 year after admission.

### Conclusion

In this systematic review and meta-analysis, orthogeriatric care had a positive effect (i.e., a reduction) on LOS, in-hospital mortality, 1-year mortality, and delirium of hip fracture patients. For LOS and TTS, the orthopedic surgeon-led model with the patient on an orthopedic ward and systematic geriatric medicine consult service (GCS) had the most consistent effect, while for in-hospital mortality, only the model with shared responsibility of the orthopedic surgeon and the geriatrician (ICM) showed a significant risk reduction. The evidence for an effect on 30-day readmission rate and functional outcome was inconsistent, while some evidence exists for less medical and surgical complications and less healthcare costs associated with orthogeriatric care. Increased awareness and routine screening, however, may explain the higher incidence of geriatric syndromes that can be observed in orthogeriatric care units. We found substantial heterogeneity and limited number of trials for some outcomes in addition to an almost complete lack of direct comparison between ICM, GCS, and GW. Therefore, adequately powered RCTs with a direct comparison between different models of care are needed to evaluate the effect of orthogeriatrics on hip fracture patients, especially with respect to 30-day readmission rate and functional outcomes.

## Supplementary Information

Below is the link to the electronic supplementary material.

Supplementary file1 (DOCX 30 kb) Search strings
