# Supplementary material for: Effects of Orthogeriatric Care Models on Outcomes of Hip Fracture Patients: A Systematic Review and Meta-Analysis
Source: Calcif Tissue Int. 2021 Sep 30;110(2):162–84. doi: 10.1007/s00223-021-00913-5 (PMC8784368; doi:10.1007/s00223-021-00913-5)
Supplement: Supplementary file 1 — Supplementary file1 (DOCX 30 kb) Search strings [file 223_2021_913_MOESM1_ESM.docx]

# Supplementary data S1: Search strings

1. Pubmed

("Patient Care Planning"[Mesh] OR Patient-Care-Planning*[tiab] OR Nursing-Care-Plan*[tiab] OR Care-Goal*[tiab] OR “Orthopedic Surgeons”[Mesh] OR Orthopedic-Surgeon*[tiab] OR Orthopedist*[tiab] OR "Geriatricians"[Mesh] OR Geriatrician*[tiab] OR Gerontologist*[tiab] OR "Interdisciplinary Communication"[Mesh] OR Interdisciplinary-Communication*[tiab] OR Multidisciplinary-Communication*[tiab] OR Cross-Disciplinary-Communication*[tiab] OR "Referral and Consultation"[Mesh:NoExp] OR Referral-and-Consultation*[tiab] OR Health-Service-Gatekeeper*[tiab] OR Referral*[tiab] OR Consultation*[tiab] OR Hospital-Referral*[tiab] OR "Patient Care Team"[Mesh] OR Patient-Care-Team*[tiab] OR Medical-Care-team*[tiab] OR Interdisciplinary-Health-Team*[tiab] OR Health-Care-Team*[tiab] OR "Transfer Agreement"[Mesh] OR Transfer-Agreement*[tiab] OR "Models, Organizational"[Mesh] OR organizational-model*[tiab] OR organisational-model*[tiab] OR "Comprehensive Health Care"[Mesh:NoExp] OR "Primary Health Care"[Mesh:NoExp] OR comprehensive-health-care[tiab] OR primary-health-care[tiab] OR primary-healthcare[tiab] OR primary-care[tiab] OR "Health Services for the Aged"[Mesh] OR “health services for the aged”[tiab] OR “health service for the aged”[tiab] OR geriatric-health-service*[tiab] OR “health services for the elderly”[tiab] OR “health services for aged”[tiab] OR Orthogeriatric*[tiab] OR care-model*[tiab] OR “models of care”[tiab] OR "Geriatric fracture center"[tiab] OR “Shared care”[tiab] OR Fracture-liaison-service*[tiab] OR “FLS”[tiab] OR "GLS"[tiab] OR Collaborative-management*[tiab] OR Care-management*[tiab] OR "Collaborative care"[tiab] OR comanagement*[tiab] OR co-management*[tiab] OR "team approach"[tiab] OR "protocol driven"[tiab] OR "integrated care"[tiab] OR "multidisciplinary care"[tiab] OR multidisciplinary-care-model*[tiab] OR multidisciplinary-management*[tiab] OR "Health care policy"[tiab] OR "Health care delivery"[tiab] OR Process-management*[tiab] OR clinical-protocol*[tiab]) AND ("Osteoporosis"[Mesh] OR “osteoporosis”[tiab] OR “osteoporoses”[tiab] OR “osteoporotic”[tiab] OR "age related bone loss"[tiab] OR "age related bone losses"[tiab] OR "Osteoporotic Fractures"[Mesh] OR osteoporotic-fracture*[tiab] OR "Femoral Fractures"[Mesh] OR femoral-fracture*[tiab] OR hip-fracture*[tiab] OR femoral-head-fracture*[tiab] OR femoral-neck-fracture*[tiab] OR femur-neck-fracture*[tiab] OR femur-head-fracture*[tiab] OR “subtrochanteric”[tiab] OR “intertrochanteric”[tiab] OR “trochanteric”[tiab] OR "Bone Diseases, Metabolic"[Mesh:NoExp] OR osteopenia*[tiab] OR metabolic-bone-disease*[tiab])

1. Embase

('osteoporosis'/de OR 'osteoporosis':ti,ab,kw OR 'decalcification':ti,ab,kw OR 'endocrine-osteoporosis':ti,ab,kw OR 'osteoporotic-decalcification':ti,ab,kw OR 'postmenopause-osteoporosis'/exp OR 'postmenopause osteoporosis':ti,ab,kw OR 'postmenopausal osteoporosis':ti,ab,kw OR 'senile osteoporosis'/exp OR 'senile osteoporosis':ti,ab,kw OR 'osteoporosis senilis':ti,ab,kw OR 'involutional-osteoporosis'/exp OR 'involutional-osteoporosis':ti,ab,kw OR 'primary-osteoporosis'/exp OR 'primary-osteoporosis':ti,ab,kw OR 'secondary osteoporosis'/exp OR 'secondary osteoporosis':ti,ab,kw OR 'bone demineralization'/exp OR 'bone demineralization':ti,ab,kw OR 'bone decalcification':ti,ab,kw OR 'osteoporotic hip fracture' OR 'osteoporotic hip fracture':ti,ab,kw OR 'fragility fracture'/exp OR 'fragility fracture*':ti,ab,kw OR 'osteoporotic fracture*':ti,ab,kw OR 'femur fracture'/exp OR 'femoral fracture*':ti,ab,kw OR 'femur torsion fracture':ti,ab,kw OR 'hip fracture'/exp OR 'hip fracture*':ti,ab,kw OR 'osteopenia'/de) AND ('patient care planning'/exp OR 'patient care planning':ti,ab,kw OR 'patient care plan':ti,ab,kw OR 'orthopedic surgeon'/exp OR 'orthopaedic surgeon':ti,ab,kw OR 'orthopedic surgeons':ti,ab,kw OR 'geriatrician'/exp OR 'geriatrician*':ti,ab,kw OR 'geriatric care'/exp OR 'geriatric care':ti,ab,kw OR 'gerontologic care':ti,ab,kw OR 'nursing care plan'/exp OR 'nursing care plan':ti,ab,kw OR 'interdisciplinary communication'/exp OR 'interdisciplinary communication':ti,ab,kw OR 'cross-disciplinary communication':ti,ab,kw OR 'inter-disciplinary communication':ti,ab,kw OR 'multi-disciplinary communication':ti,ab,kw OR 'multidisciplinary communication':ti,ab,kw OR 'trans-disciplinary communication':ti,ab,kw OR 'transdisciplinary communication':ti,ab,kw OR 'patient referral'/exp OR 'patient referral':ti,ab,kw OR 'gatekeeping':ti,ab,kw OR 'referral':ti,ab,kw OR 'referral and consultation':ti,ab,kw OR 'continuity of care':ti,ab,kw OR 'continuity of patient care':ti,ab,kw OR 'patient care management':ti,ab,kw OR 'patient care team':ti,ab,kw OR 'patient management':ti,ab,kw OR 'patient navigation':ti,ab,kw OR 'comprehensive health care':ti,ab,kw OR 'primary health care'/exp OR 'primary health care':ti,ab,kw OR 'health services for the aged':ti,ab,kw OR 'orthogeriatrics'/exp OR 'orthogeriatrics':ti,ab,kw OR 'shared care'/exp OR 'shared care':ti,ab,kw OR 'fracture liaison service'/exp OR 'fracture liaison service':ti,ab,kw OR 'collaborative care team'/exp OR 'collaborative care team':ti,ab,kw OR 'collaborative health care team':ti,ab,kw OR 'collaborative healthcare team':ti,ab,kw OR 'collaborative patient care team':ti,ab,kw OR 'integrated care'/exp OR 'integrated care':ti,ab,kw OR 'integrated health care system'/exp OR 'integrated health care system':ti,ab,kw OR 'delivery of health care, integrated':ti,ab,kw OR 'integrated health care delivery':ti,ab,kw OR 'integrated health care delivery system':ti,ab,kw OR 'multidisciplinary team'/exp OR 'multidisciplinary team':ti,ab,kw OR 'multidisciplinary care'/exp OR 'multidisciplinary care':ti,ab,kw OR 'multidisciplinary management'/exp OR 'multidisciplinary management':ti,ab,kw OR 'multidisciplinary approach'/exp OR 'multidisciplinary approach':ti,ab,kw OR 'clinical protocol'/exp OR 'clinical protocol*':ti,ab,kw OR 'clinical research protocol':ti,ab,kw)

1. Web of science

('osteoporosis'/de OR 'osteoporosis':ti,ab,kw OR 'decalcification':ti,ab,kw OR 'endocrine-osteoporosis':ti,ab,kw OR 'osteoporotic-decalcification':ti,ab,kw OR 'postmenopause-osteoporosis'/exp OR 'postmenopause osteoporosis':ti,ab,kw OR 'postmenopausal osteoporosis':ti,ab,kw OR 'senile osteoporosis'/exp OR 'senile osteoporosis':ti,ab,kw OR 'osteoporosis senilis':ti,ab,kw OR 'involutional-osteoporosis'/exp OR 'involutional-osteoporosis':ti,ab,kw OR 'primary-osteoporosis'/exp OR 'primary-osteoporosis':ti,ab,kw OR 'secondary osteoporosis'/exp OR 'secondary osteoporosis':ti,ab,kw OR 'bone demineralization'/exp OR 'bone demineralization':ti,ab,kw OR 'bone decalcification':ti,ab,kw OR 'osteoporotic hip fracture' OR 'osteoporotic hip fracture':ti,ab,kw OR 'fragility fracture'/exp OR 'fragility fracture*':ti,ab,kw OR 'osteoporotic fracture*':ti,ab,kw OR 'femur fracture'/exp OR 'femoral fracture*':ti,ab,kw OR 'femur torsion fracture':ti,ab,kw OR 'hip fracture'/exp OR 'hip fracture*':ti,ab,kw OR 'osteopenia'/de) AND ('patient care planning'/exp OR 'patient care planning':ti,ab,kw OR 'patient care plan':ti,ab,kw OR 'orthopedic surgeon'/exp OR 'orthopaedic surgeon':ti,ab,kw OR 'orthopedic surgeons':ti,ab,kw OR 'geriatrician'/exp OR 'geriatrician*':ti,ab,kw OR 'geriatric care'/exp OR 'geriatric care':ti,ab,kw OR 'gerontologic care':ti,ab,kw OR 'nursing care plan'/exp OR 'nursing care plan':ti,ab,kw OR 'interdisciplinary communication'/exp OR 'interdisciplinary communication':ti,ab,kw OR 'cross-disciplinary communication':ti,ab,kw OR 'inter-disciplinary communication':ti,ab,kw OR 'multi-disciplinary communication':ti,ab,kw OR 'multidisciplinary communication':ti,ab,kw OR 'trans-disciplinary communication':ti,ab,kw OR 'transdisciplinary communication':ti,ab,kw OR 'patient referral'/exp OR 'patient referral':ti,ab,kw OR 'gatekeeping':ti,ab,kw OR 'referral':ti,ab,kw OR 'referral and consultation':ti,ab,kw OR 'continuity of care':ti,ab,kw OR 'continuity of patient care':ti,ab,kw OR 'patient care management':ti,ab,kw OR 'patient care team':ti,ab,kw OR 'patient management':ti,ab,kw OR 'patient navigation':ti,ab,kw OR 'comprehensive health care':ti,ab,kw OR 'primary health care'/exp OR 'primary health care':ti,ab,kw OR 'health services for the aged':ti,ab,kw OR 'orthogeriatrics'/exp OR 'orthogeriatrics':ti,ab,kw OR 'shared care'/exp OR 'shared care':ti,ab,kw OR 'fracture liaison service'/exp OR 'fracture liaison service':ti,ab,kw OR 'collaborative care team'/exp OR 'collaborative care team':ti,ab,kw OR 'collaborative health care team':ti,ab,kw OR 'collaborative healthcare team':ti,ab,kw OR 'collaborative patient care team':ti,ab,kw OR 'integrated care'/exp OR 'integrated care':ti,ab,kw OR 'integrated health care system'/exp OR 'integrated health care system':ti,ab,kw OR 'delivery of health care, integrated':ti,ab,kw OR 'integrated health care delivery':ti,ab,kw OR 'integrated health care delivery system':ti,ab,kw OR 'multidisciplinary team'/exp OR 'multidisciplinary team':ti,ab,kw OR 'multidisciplinary care'/exp OR 'multidisciplinary care':ti,ab,kw OR 'multidisciplinary management'/exp OR 'multidisciplinary management':ti,ab,kw OR 'multidisciplinary approach'/exp OR 'multidisciplinary approach':ti,ab,kw OR 'clinical protocol'/exp OR 'clinical protocol*':ti,ab,kw OR 'clinical research protocol':ti,ab,kw)
